# Knockout of LASP1 in CXCR4 expressing CML cells promotes cell persistence, proliferation and TKI resistance

**DOI:** 10.1111/jcmm.14910

**Published:** 2020-01-19

**Authors:** Andreas B. Herrmann, Martha‐Lena Müller, Martin F. Orth, Jörg P. Müller, Alma Zernecke, Andreas Hochhaus, Thomas Ernst, Elke Butt, Jochen J. Frietsch

**Affiliations:** ^1^ Institut für Experimentelle Biomedizin II Universitätsklinikum Würzburg Würzburg Germany; ^2^ Rudolf‐Virchow‐Zentrum für Experimentelle Biomedizin I Universitätsklinikum Würzburg Würzburg Germany; ^3^ Labor für Pädiatrische Sarkombiologie Medizinische Fakultät Pathologisches Institut LMU München München Germany; ^4^ Center for Molecular Biomedicine Institut für Molekulare Zellbiologie Universitätsklinikum Jena Jena Germany; ^5^ Abteilung für Hämatologie und internistische Onkologie Klinik für Innere Medizin II Universitätsklinikum Jena Jena Germany

**Keywords:** BCR‐ABL, CML, CXCR4, LASP1, nilotinib, precursor cells

## Abstract

Chronic myeloid leukaemia (CML) is a clonal myeloproliferative stem cell disorder characterized by the constitutively active BCR‐ABL tyrosine kinase. The LIM and SH3 domain protein 1 (LASP1) has recently been identified as a novel BCR‐ABL substrate and is associated with proliferation, migration, tumorigenesis and chemoresistance in several cancers. Furthermore, LASP1 was shown to bind to the chemokine receptor 4 (CXCR4), thought to be involved in mechanisms of relapse. In order to identify potential LASP1‐mediated pathways and related factors that may help to further eradicate minimal residual disease (MRD), the effect of LASP1 on processes involved in progression and maintenance of CML was investigated. The present data indicate that not only overexpression of CXCR4, but also knockout of LASP1 contributes to proliferation, reduced apoptosis and migration as well as increased adhesive potential of K562 CML cells. Furthermore, LASP1 depletion in K562 CML cells leads to decreased cytokine release and reduced NK cell‐mediated cytotoxicity towards CML cells. Taken together, these results indicate that in CML, reduced levels of LASP1 alone and in combination with high CXCR4 expression may contribute to TKI resistance.

## 1 INTRODUCTION

Over the past decade, tyrosine kinase inhibitor (TKI) therapy has transformed chronic myeloid leukaemia (CML), caused by the BCR‐ABL fusion‐oncoprotein,^1^ from a fatal disease into a chronic ailment.^2^ Actually, survival of CML patients is mainly determined by comorbidities rather than by the disease itself.^1^, ^3^, ^4^ Hence, for CML patients with optimal response to TKIs and long‐term survival, treatment‐free remission (TFR) has become a therapeutic goal. However, the STIM, TWISTER and EURO‐SKI trials estimated a 2‐year molecular remission probability of only 38%, 47% and 52% after TKI withdrawal, respectively.^5^, ^6^, ^7^

Further, there are still patients who lack deep molecular response (DMR), develop resistances or even progress into accelerated or blast phase. For patients with optimal response, loss of major molecular response (MMR) is considered to be a reliable threshold for re‐administration of TKI^1^ during the treatment‐free period. Routine molecular monitoring for minimal residual disease (MRD) during TKI treatment cannot serve as a reliable clinical test to identify patients who can safely discontinue their TKI treatment without risk of a molecular relapse.

A proposed mechanism for molecular relapse in CML patients involves leukaemic stem cell homing to the bone marrow, mediated through the chemokine receptor 4 (CXCR4)‐C‐X‐C motif chemokine 12 (CXCR12) axis. While BCR‐ABL expression has been reported to down‐regulate CXCR4 levels, TKI treatment was shown to restore the chemokine receptor's surface expression. Subsequent activation of the receptor by CXCL12 leads to homing of leukaemic stem cells (LSCs) to the bone marrow (BM) microenvironment, which is thought to promote quiescence and survival of the cells. After treatment discontinuation, these surviving CML stem cells may cause a relapse of the disease.^8^

A recently identified CXCR4 binding partner and overexpressed substrate of BCR‐ABL in CML is LASP1.^9^ The protein has initially been identified from a cDNA library of breast cancer metastases. LASP1 is overexpressed in several human tumours and is involved in cell proliferation, migration, tumorigenesis and chemoresistance.^10^, ^11^ The protein comprises an N‐terminal LIM domain, two nebulin‐like actin binding repeats, a linker region with two phosphorylation sites at S146 and Y171 and a C‐terminal SH3 domain.^12^ Furthermore, LASP1 has been identified as member of a six genes signature being highly predictive for CML disease phases, thereby allowing a more precise prediction of relapse after stem cell transplantation than clinical risk factors alone.^13^

Binding of LASP1 to the conserved leucine/isoleucine (LKIL) motif at the carboxy‐terminal domain of CXCR4 requires phosphorylation of LASP1 at S146 and stabilizes the chemokine receptor.^14^, ^15^ In breast cancer cells, receptor activation then results in the translocation of LASP1 to the nucleus and interference of the protein with the epigenetic machinery.^16^

In CML cells, however, the function and effects of LASP1 have not been investigated. The present work now demonstrates that low LASP1 levels, as observed in TKI non‐responders and blast crisis patients, affect proliferation, migration and cytokine release in the CML cell line K562 and might contribute to worse patient outcome.

## 2 PATIENTS, MATERIALS AND METHODS

### 2.1 Cell culture

K562 cells were purchased from ATCC and HUVEC from PromoCell. NK‐92C cells were a kind gift from Prof. Dr rer. nat. Carsten Watzl, Leibniz Research Centre for Working Environment and Human Factors (IfADo, TU‐Dortmund).

### 2.2 Microarray and gene set enrichment analyses

Publicly available gene expression data of n = 62 individual CML patients were retrieved from the Gene Expression Omnibus (GEO) and the Array Express platform hosted at the EBI (http://www.ebi.ac.uk/arrayexpress/). Accession numbers GSE14671: CD34‐positive selected BM cells of n = 59 patients in late or newly diagnosed chronic phase 17 and GSE48294: CD34+ selected cells from n = 3 patients in chronic phase cultured in vitro with or without imatinib under normoxia (21% O_2_) or hypoxia (0.5% O_2_) for 24 or 96 hours.^18^ Data with accession number GSE14671 were generated on Affymetrix HG‐U133plus2.0 microarrays and with the accession number GSE48294 on Illumina HT‐12 beadchips. All CEL files of the GSE14671 data set were simultaneously normalized with robust multi‐array average (RMA)^19^ using a custom brain array chip description file (CDF; v20 ENTREZG).^20^ For the analysis of the GSE48294 data set, already normalized expression values from the series matrix file were used.

For interpretation of biological pathways correlated with LASP1 expression in patients with response to imatinib treatment (n = 41) vs non‐responders (n = 18; GSE14671),^17^ a gene set analysis (GSEA) was performed.^21^ Pre‐ranked GSEA was carried out for both groups utilizing lists of all genes in the data set ranked by their Pearson correlation coefficient with *LASP1* expression, and 4762 curated gene sets downloaded from the Broad Institute (Cambridge, MA, USA; http://software.broadinstitute.org/gsea/msigdb/index.jsp; c2.all.v5.1). To assess significance, the analyses were repeated with 1000 permutations of the pre‐ranked gene lists. Results were analysed, respecting the normalized enrichment score (NES), *P*‐value corrected for multiple testing (FWER) and false discovery rate (FDR).

### 2.3 Generation of LASP1 knockout cells

**Figure 1:**
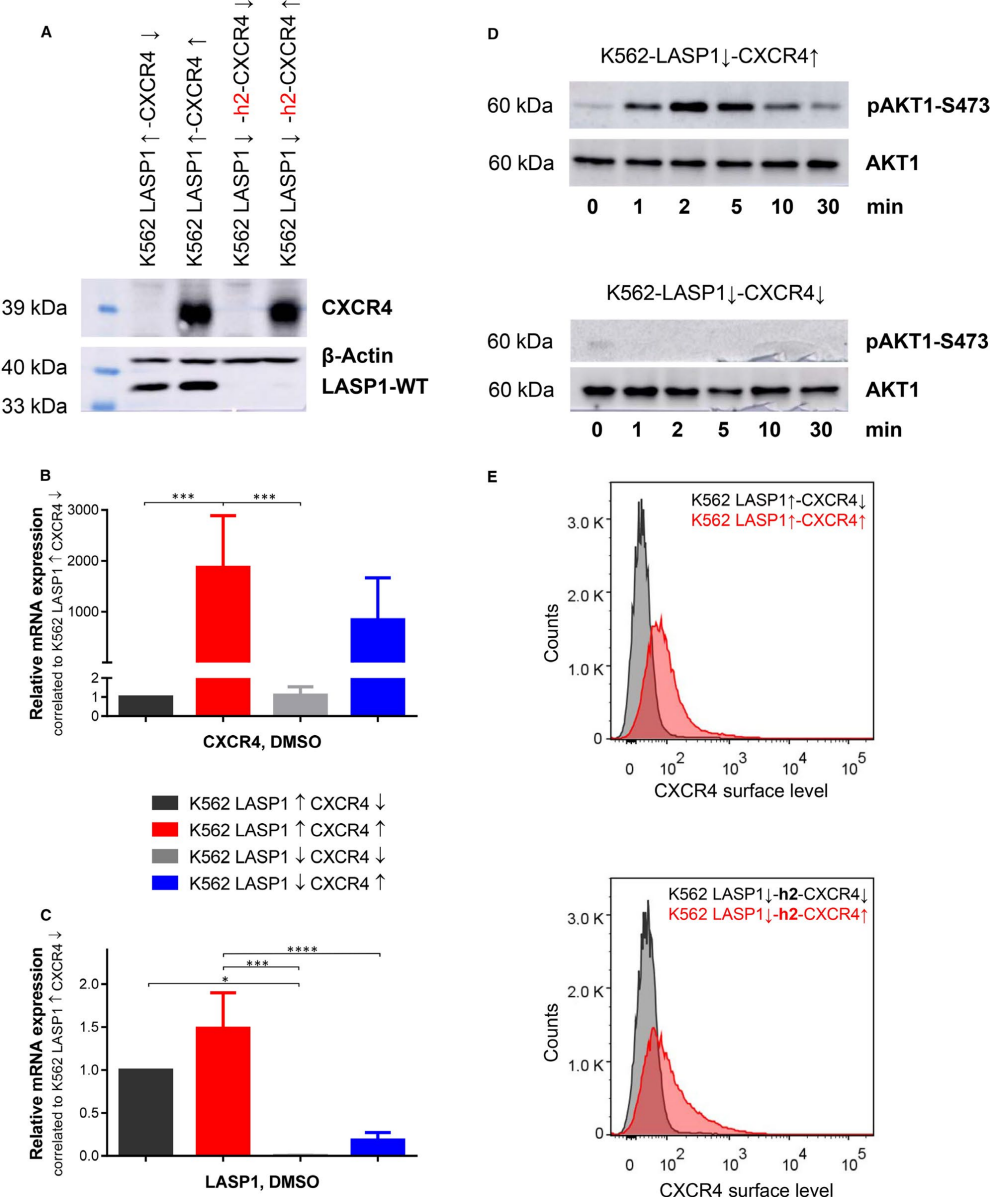
Validation of K562 cells with inactivated LASP1 and CXCR4 overexpression. A, Western blot analysis (10% gel) of LASP1 and CXCR4 in K562 cells after CRISPR/Cas9‐mediated LASP1 knockout and after lentiviral transduced CXCR4 expression of indicated cell lines. Relative expression of CXCR4 mRNA (B) and of LASP1 mRNA (C) in the generated cell lines was analysed by qRT‐PCR as described in the Materials and Methods section. Results represent the mean of three independent experiments ± SD. D, CXCR4 functionality in CXCR4 overexpressing K562 cells was validated by AKT1‐S473 phosphorylation after 25 nmol/L (200 ng/mL) CXCL12 stimulation in a time dependent manner by Western blot analysis (10% gel). β‐actin served as loading control. E, Flow cytometric analysis of CXCR4 surface expression (50 000 recorded events). CXCR4 cell surface expression in CXCR4 overexpressing cell lines exceeded non‐transduced cell lines. CXCL12, C‐X‐C motif chemokine 12; CXCR4, chemokine receptor 4; DMSO, dimethyl sulfoxide; LASP1, LIM and SH3 domain protein 1; SD, standard deviation

CRISPR/Cas9 LASP1 plasmids (SC‐404630‐NIC and SC‐404630‐NIC‐2) were purchased from Santa Cruz (Heidelberg, Germany). A plasmid encoding a non‐targeting gRNA was used as negative control (SC‐437281; Santa Cruz). UltraCruz^®^ Transfection Reagent (SC‐395739; Santa Cruz) and Plasmid Transfection Medium (SC‐108062; Santa Cruz) were used for transfection. Transfection of K562 cells was performed according to the manufacturer's instruction. Detailed protocols for the generation of LASP1 knockout cells are described in Supplemental Materials and Methods. The double selected cells were then seeded into methylcellulose in the absence of cytokines (MethoCult H4230; STEMCELL Technologies SARL) in order to receive clonal cell cultures. Colonies were counted after 7‐14 days of incubation and finally transferred back into liquid culture. Complete biallelic knockout of LASP1 was checked by Western blot (Figure 1A) and qRT‐PCR (Figure 1C). Sanger sequencing demonstrated deletion of 32 base pairs (Figure S9). In each case, 5 clones were pooled to establish K562‐LASP1−/− (LASP knockout) and K562‐LASP1+/+ (control plasmid) cell lines, cultivated in RPMI + GlutaMAX‐1 (Gibco™, Thermo Fisher) with 10% FCS (Biochrom, Berlin, Germany). Wild‐type cells transfected with control plasmid will be referred to as K562‐LASP1↑, whereas cells with the LASP1 knockout plasmids SC‐404630‐NIC and SC‐404630‐NIC2 are referred to as K562‐LASP1↓‐h1 and K562‐LASP1↓‐h2, respectively. Sustained sensitivity to puromycin was checked.

### 2.4 Generation of CXCR4 expressing cell lines

For generation of lentiviral particles, human embryonic kidney 293T (HEK293T) cells were cultivated in Dulbecco's modified Eagle's medium (DMEM; Gibco™) supplemented with 10% FCS. The cells were transiently transfected with the CXCR4‐encoding pCDH‐CMV‐MCS‐EF1‐Puro plasmid in combination with viral packaging vectors (pMDL, pRSV and pVSV‐G; all Addgene Europe), using polyethyleneimine (PEI; Sigma‐Aldrich). Detailed information about the cloning of CXCR4 can be found in Supplemental Material and Methods. Lentivirus‐containing supernatant was collected 24, 48 and 72 hours after transfection. K562‐LASP1↓‐h1/h2 and K562‐LASP1↑ cells were immediately infected three times with the pseudotyped particles in the presence of 8 µg/mL polybrene (1,5‐dimethyl‐1,5‐diazaundecamethylene polymethobromide, AL‐118, Sigma‐Aldrich). Transduction efficiency was 90%‐95% as assessed by parallel control transduction with GFP‐expressing viral particles. Selection was carried out using 2 µg/mL puromycin. Efficiency of shRNA‐mediated LASP1 knockout was checked by Western blot. Cell surface expression of CXCR4 was analysed by flow cytometry using BD FACSCalibur™ and BD CellQuest™ software (Becton Dickinson). PE anti‐human CD184 (CXCR4) antibody and PE mouse IgG2a (κ isotype control antibody) were purchased from BioLegend. Only transfection of K562‐LASP1↓‐h2 was successful (Figure 1A). The following cell lines were generated and cultivated in RPMI with 10% FCS: K562‐LASP1↑‐CXCR4↑, K562‐LASP1↑‐CXCR4↓, K562‐LASP1↓‐CXCR4↑ and K562‐LASP1↓‐CXCR4↓.

### 2.5 Migration assay

For migration assays, Corning^®^ FluoroBlok™ Cell Culture Inserts with a pore size of 8 μm combined with 24‐well cell culture plates were used. Before start of the migration assay, the cells were serum‐starved overnight in RPMI with 1% BSA. Cells were then stained with CellTracker™ Red CMPTX dye (Thermo Fisher) at a final concentration of 10 µM for 30 minutes in serum‐free RPMI without phenol red. Cells were pelleted and again incubated in serum‐free RPMI for 30 minutes without phenol red to remove the surplus dye. Cells were seeded in duplets onto the insert membrane at a density of 3 × 10^5^ cells/300 µL per insert. The bottom compartment was filled with 1.0 mL of the same medium. After 30 minutes rest period, cells were placed into wells containing the specified chemoattractant and were allowed to migrate through the membrane for 48 hours. Migration was quantified from the bottom of the wells by fluorescence measurement using the CLARIOstar microplate reader (BMG; Extinction: 577 nm; Emission: 607 nm) every 10 minutes within the first two hours and then every 30 minutes for 46 hours. Cell migration was displayed as signal compared to the measurement at 4 hours.

### 2.6 Adhesion assay

Adhesion assays under flow conditions were performed as previously described.^22^ Detailed information can be found in Supplemental Materials and Methods.

### 2.7 Degranulation and cytotoxicity assay

Degranulation response of NK‐92C to K562 cells was assessed by flow cytometric analysis of LAMP1 (CD107a) surface expression after 4 hours co‐culture. Assays were performed at 2:1, 1:1 and 0.5:1 effector to target ratios in the presence of APC‐labelled anti‐human CD107a antibody (BioLegend). Subsequently, NK cells were stained with PerCP/Cy5.5 anti‐human CD56 antibodies (BioLegend) followed by flow cytometric analysis.^23^

### 2.8 Detection of cytokines and chemokines

Basal release of IL‐1β, IFN‐α2, IFN‐γ, TNF‐α, MCP‐1 (CCL2), IL‐6, IL‐8 (CXCL8), IL‐10, IL‐12p70, IL‐17A, IL‐18, IL‐23 and IL‐33 was quantified in the conditional medium after 4‐h and 24‐h culture of 1 × 10^6^ K562 cells/mL, respectively, 5 × 10^5^ K562 cells/mL, using the Human Inflammation Panel (LEGENDplex™, BioLegend) bead‐based immunoassay according to manufacturer's instructions. Samples were analysed with a BD FACSCalibur™ using the BD CellQuest™ software (Becton Dickinson) and LEGENDplex™ Data Analysis Software. Cytokine release quantification was adjusted to the cell number.

### 2.9 Patients and healthy controls

We additionally analysed a cohort of 57 patients (13 female; median age 55 years, range 19‐76; 18 patients with transcript b2a2, 26 with b3a2 and 13 with b2a2 as well as b3a2; median EUTOS‐Score 28, range 0‐196) newly diagnosed to suffer from CML in chronic phase at the university hospital of Jena, Thuringia, Germany between September 2012 and July 2015. The study has been approved by the institutional ethics committee and patients provided written informed consent in accordance with the Declaration of Helsinki.

### 2.10 Statistics

Unless otherwise indicated, all experiments were performed in three independent experiments in triplicates and results are expressed as means ± standard deviation (SD). Statistical comparison of means was performed by ANOVA using GraphPad Prism 8.0.2 (GraphPad Inc). Wilcoxon‐Mann‐Whitney test was used to compare patients' cohort with control group. Data were considered to be significant with *P* < .05 (*P < .05; **P < .01; ***P < .001; ****P < .0001).

Additional Supplemental Materials and Methods can be found in Supplemental Information.

## 3 RESULTS

### 3.1 LASP1 expression is lower in imatinib non‐responders and inversely regulated with gene sets associated with ATP generation, transcription and translation

Microarray analyses of whole BM probes from CML patients in chronic phase, accelerated phase and blast crisis, analysed by Yeung and colleagues, have demonstrated that the protein LASP1 is a member of a six genes signature which is highly predictive for CML disease phases and allows a precise prediction of relapse after stem cell transplantation.^13^ While the association between certain expression patterns and disease progression as well as therapy response could be identified in CD34 selected blood and bone marrow samples, this was not possible in unselected probes.^17^, ^24^ For that reason, we used microarray data of isolated CD34‐positive BM cells in order to further assess the diagnostic value of LASP1 for patient outcome. The clinicopathological data allowed for discrimination between TKI responders (n = 41) and non‐responders (n = 18). Within a set of n = 36 samples of CML patients in the late chronic phase and n = 23 samples of newly diagnosed chronic phase patients,^17^ *LASP1*, *S100P*, *SFN* and *CXCR4* mRNA were found to be expressed significantly lower (*P* < .05; marked by colour) in patients' samples not responding to imatinib therapy (Table 1). Here, LASP1 was positively correlated with *CRKL* and *STAT3* but negatively correlated with *S100A4* and *ZO‐2*. In imatinib responders, a positive correlation of LASP1 with *LYN*, *S100A11*, *S100P*, *MMP9*, *SFN*, *CXCR2*, *LPP*, *PALLD*, *VASP*, *ZYX, CD34*, *STAT3* and *DNMT3A* was observed, while *LASP1* showed a negative correlation with *SOX9*, *PPP3CC* and *COPS5* (Table 1).

**Table 1:** Expression and correlation of LASP1 and LASP1 binding and interaction partners in imatinib responders vs non‐responders

Analysis based on GSE14671						
Gene	FC (responders vs. Non‐responders)	*P*‐value (*t* test)	Non‐responders		Responders	
			Correlation with LASP1	*P*‐value	Correlation with LASP1	*P*‐value
**LASP1**	**1.1713**	**.0017**				
HIF1alpha	1.1263	.2629	−0.0002	.9992	0.0762	.6360
SOX9	1.0322	.1380	0.0043	.9866	***−0.3197***	***.0416***
SRC	1.0137	.6638	0.0688	.7862	0.2319	.1445
LYN	1.0066	.9396	0.3721	.1284	***0.4579***	***.0026***
PRKACA	1.0522	.2327	0.4039	.0965	0.2960	.0603
PRKACG	1.0550	.1284	0.2561	.3051	0.0855	.5951
**PRKG1**	**0.8053**	**.0134**	−0.1731	.4921	0.1650	.3026
PPP3CA	0.8704	.0577	−0.2144	.3929	−0.1630	.3085
PPP3CB	0.8731	.1114	0.0707	.7804	0.0489	.7615
PPP3CC	1.0531	.5979	−0.2479	.3213	***−0.4941***	***.0010***
S100A4	0.9890	.9290	−*0.5715*	*.0132*	−0.2226	.1618
S100A11	1.3976	.0571	−0.1129	.6555	***0.3210***	***.0407***
**S100P**	**2.3839**	**.0259**	0.3213	.1935	***0.5266***	***.0004***
MMP1	1.0018	.9495	0.3949	.1049	0.1168	.4669
MMP3	1.0235	.3815	−0.0493	.8459	−0.0330	.8375
MMP9	1.9821	.1528	0.3866	.1131	***0.5570***	***.0002***
COPS5	0.9993	.9926	−0.0794	.7541	***−0.5585***	***.0001***
**SFN**	**1.2045**	**.0099**	0.3843	.1153	***0.3164***	***.0439***
PTEN	0.9171	.2193	0.0425	.8671	0.1044	.5158
CXCR2	1.3280	.1960	0.2946	.2354	***0.4516***	***.0030***
KLHL41	1.0325	.3629	0.0977	.6997	0.0775	.6300
DNM2	1.0937	.2208	−0.3538	.1498	−0.2511	.1133
LPP	0.9836	.7442	0.3915	.1081	***0.4671***	***.0021***
PALLD	1.1015	.2163	0.0731	.7731	***0.4698***	***.0019***
VASP	1.1433	.1783	0.0104	.9672	***0.4639***	***.0022***
VIM	1.1484	.3804	0.0409	.8720	0.2697	.0881
ZO2	0.9345	.1004	−*0.5294*	*.0239*	−0.0277	.8635
ZYX	1.0361	.7470	0.2542	.3088	***0.5552***	***.0002***
CD34	0.9125	.6016	0.3343	.1752	***0.5608***	***.0001***
**CXCR4**	**1.5743**	**.0237**	0.0707	.7804	0.1111	.4894
CRKL	1.0332	.5457	*0.6370*	*.0045*	0.0079	.9610
STAT3	1.1082	.1669	*0.5203*	*.0268*	***0.5846***	***.0001***
STAT5A	1.0427	.5439	0.4046	.0958	0.2588	.1023
STAT5B	0.9832	.7258	0.0288	.9096	−0.0050	.9755
DNMT1	0.9954	.9342	‐0.1180	.6409	‐0.2008	.2082
DNMT3A	1.0560	.4105	0.2736	.2719	***0.5486***	***.0002***
DNMT3B	0.9212	.1829	‐0.0481	.8496	‐0.1842	.2491

Note

Bold: Significantly differentially expressed in responders and non‐responders.

Italics: Significantly (counter)coregulated with LASP1 expression in non‐responders.

Bold with italics: Significantly (counter)coregulated with LASP1 expression in responders.

LASP1 interaction partners: Publicly available microarray data sets of isolated CD34‐positive BM cells were discriminated in TKI responders and non‐responders.^17^ The according CEL files were normalized with RMA^19^ using a custom brain array CDF.^20^ Genes being significantly lower expressed in patients' samples not responding to imatinib therapy are marked in green. Genes being significantly coregulated with LASP1 in responders and non‐responders are marked in yellow and orange, respectively.

Abbreviations: BM, bone marrow; CDF, chip description file; CXCR4, chemokine receptor 4; DMSO, dimethyl sulfoxide; HIF1α, hypoxia‐inducible factor 1‐alpha; LASP1, LIM and SH3 domain protein 1; RMA, robust multi‐array average; WT, wild‐type.

Gene set enrichment analysis for responders and non‐responders yielded 22, respectively, 2 gene sets with a significant positive correlation, and 164, respectively, 17 gene sets, that were negatively correlated with LASP1 (for additional information see Table S2). In imatinib non‐responders, LASP1 was inversely regulated with several gene sets associated with ATP generation as well as transcriptional and translational processes (Table S2). Similar but less pronounced results were obtained for imatinib responders. In these patients, LASP1 gene sets were associated with *CXCR4*, as well as with immune cell differentiation and function.

Since *LASP1* expression is stimulated by hypoxia‐inducible factor 1‐alpha (HIF1α),^25^ we analysed the microarray data for differential *LASP1* expression under hypoxic conditions in the BM environment.^26^, ^27^ However, we did not find a positive correlation between *LASP1* and *HIF1α* in this data set. Among genes known to be relevant for regulation and function of *LASP1*, only *CXCR2* and *DNMT3A* appeared to be up‐regulated after 96 hours of hypoxia, while *SRC* was down‐regulated (Table 1).

### 3.2 Generation of a CML precursor cell LASP1 knockout model

The BCR‐ABL‐positive cell line K562 expresses low to no detectable cell surface protein and mRNA levels of CXCR4 and CXCR7.^28^ To investigate a possible role of LASP1 in CXCR4 signalling and to characterize the effect of LASP1 on disease progression, K562 cell lines with low and elevated levels of CXCR4 expression in the presence and absence of LASP1 were generated by stable CRISPR/Cas9‐based LASP1 knockout and viral transduction of CXCR4. Western blot analysis revealed complete allelic knockout of LASP1 in K562‐LASP1↓‐CXCR4↑ and K562‐LASP1↓‐CXCR4↓ clones (Figure 1A). In order to exclude possible off‐target activity, the CRISPR/Cas9 system used in this study relies on paired nicking which has been shown to reduce off‐target activity by 50‐ to 1000‐fold.^29^ Off‐target effects were further minimized by additional pooling of 5 clones of each generated cell line. qRT‐PCR reassured CXCR4 expression below limit of detection in wild‐type K562 cells transfected with empty vector (K562‐LASP1↑‐CXCR4↓) and verified positive CXCR4 expression in K562‐LASP1↑‐CXCR4↑ and K562‐LASP1↓‐CXCR4↑ (Figure 1B) as well as LASP1 knockout in K562‐LASP1↓‐CXCR4↓ and K562‐LASP1↓‐CXCR4↑ (Figure 1C). FACS analyses confirmed a clear increase in the mean fluorescence intensity (MFI) of CXCR4 cell surface expression in K562‐LASP1↑‐CXCR4↑ and K562‐LASP1↓‐CXCR4↑ (MFI 83.25 ± 9.22 and 76.20 ± 20.95 respectively) compared to the background fluorescence of non‐CXCR4 expressing cell lines K562‐LASP1↑‐CXCR4↓ and K562‐LASP1↓‐CXCR4↓ [MFI 48.25 ± 11.73 (*P* = .035) and 37.00 ± 6.79 (*P* = .0192), respectively; Figure 1E]. To check the functional activity of the transduced CXCR4, cells were stimulated with CXCL12 in a time‐resolved experiment.^28^ Major phospho‐AKT1‐S473 phosphorylation was observed within 2 minutes, and phosphorylation declined back to basal levels within 10 minutes (Figure 1D, upper panel). This signal was absent in the non‐CXCR4 expressing control cells (Figure 1D, lower panel).

Furthermore, no rescue by LASP2 was detected, neither on mRNA (Figure S10) nor on protein level (Figure 1A; molecular weight of LASP2: 34 kD).

### 3.3 Knockout of LASP1 enhances viability in the presence of nilotinib

**Figure 2:**
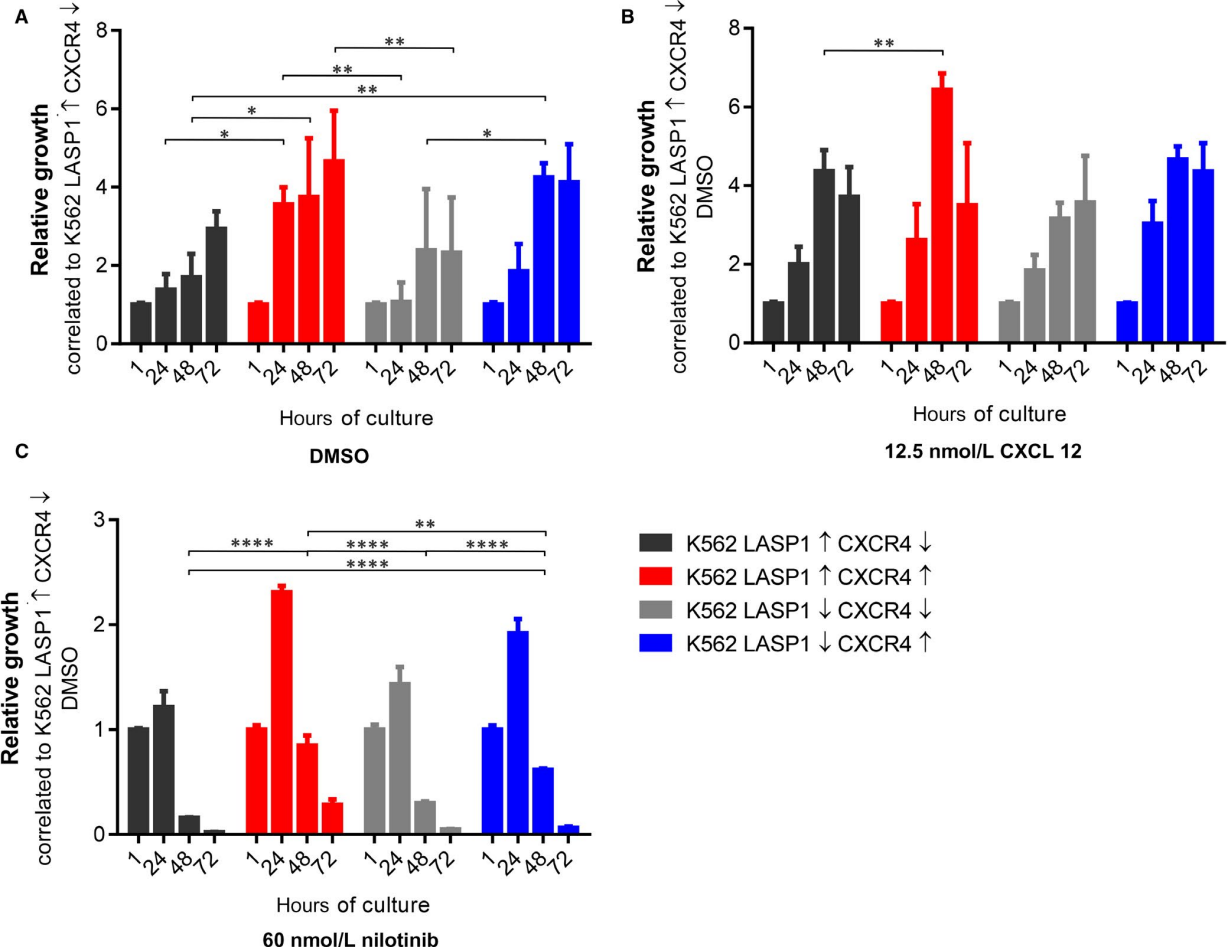
Effect of LASP1 knockout and CXCR4 overexpression on cell growth and viability. K562‐LASP1↑‐CXCR4↑, K562‐LASP1↑‐CXCR4↓, K562‐LASP1↓‐CXCR4↑ and K562‐LASP1↓‐CXCR4↓ cell lines were tested for viability using RealTime‐Glo™ MT Cell Viability Assay (Promega) according to the manufacturer's protocol in (A) DMSO control, (B) after stimulation with 12.5 nmol/L (100 ng/mL) CXCL12 and (C) after incubation with 60 nmol/L nilotinib. Results represent the mean of three independent experiments in triplicates ± SD. CXCL12, C‐X‐C motif chemokine 12; CXCR4, chemokine receptor 4; DMSO, dimethyl sulfoxide; LASP1, LIM and SH3 domain protein 1; SD, standard deviation

To investigate a possible role of LASP1 in the context of CXCR4‐mediated cell proliferation in CML, viability of the newly generated cell lines was tested under several conditions. In order to reflect clinical practice, experiments were conducted with nilotinib. This TK inhibitor has been shown to achieve faster and deeper molecular response, which is associated with a higher probability for TFR.^30^, ^31^ We used three different concentrations (30, 60 or 120 nmol/L) and stimulated with either 12.5 nmol/L CXCL12 alone or in combination with 120 nmol/L nilotinib. As an additional setting, CXCL12 stimulation was carried out after 1 hour pre‐incubation with the CXCR4 inhibitor plerixafor (Figure S1). Under basal conditions (DMSO), overexpression of CXCR4 promoted cell proliferation (K562‐LASP1↑‐CXCR4↓ and K562‐LASP1↑‐CXCR4↑, Figure 2A) independently of LASP1 level (K562‐LASP1↑‐CXCR4↑ and K562‐LASP1↓‐CXCR4↑, Figure 2A), while LASP1 knockout resulted in reduced cell growth at 24 hours (K562‐LASP1↑‐CXCR4↑ and K562‐LASP1↓‐CXCR4↑, Figure 2A). Similar effects were observed when stimulated with CXCL12 (Figure 2B). In the presence of nilotinib, all cell lines showed high sensitivity to the TKI but the pro‐proliferative effect of CXCR4 expression was still observable (K562‐LASP1↑‐CXCR4↓ and K562‐LASP1↑‐CXCR4↑, Figure 2C). However, in contrast to basal conditions, LASP1 depletion resulted in enhanced cell growth. The effect was most prominent after 24 hours in the presence of 60 nmol/L nilotinib and was still visible after 48 hours (Figure 2C; focus on blue bars). After 72 hours, nilotinib‐treated cells were dying.

### 3.4 The combination of CXCR4 overexpression and LASP1 knockout promotes resistance to nilotinib in CML cells

**Figure 3:**
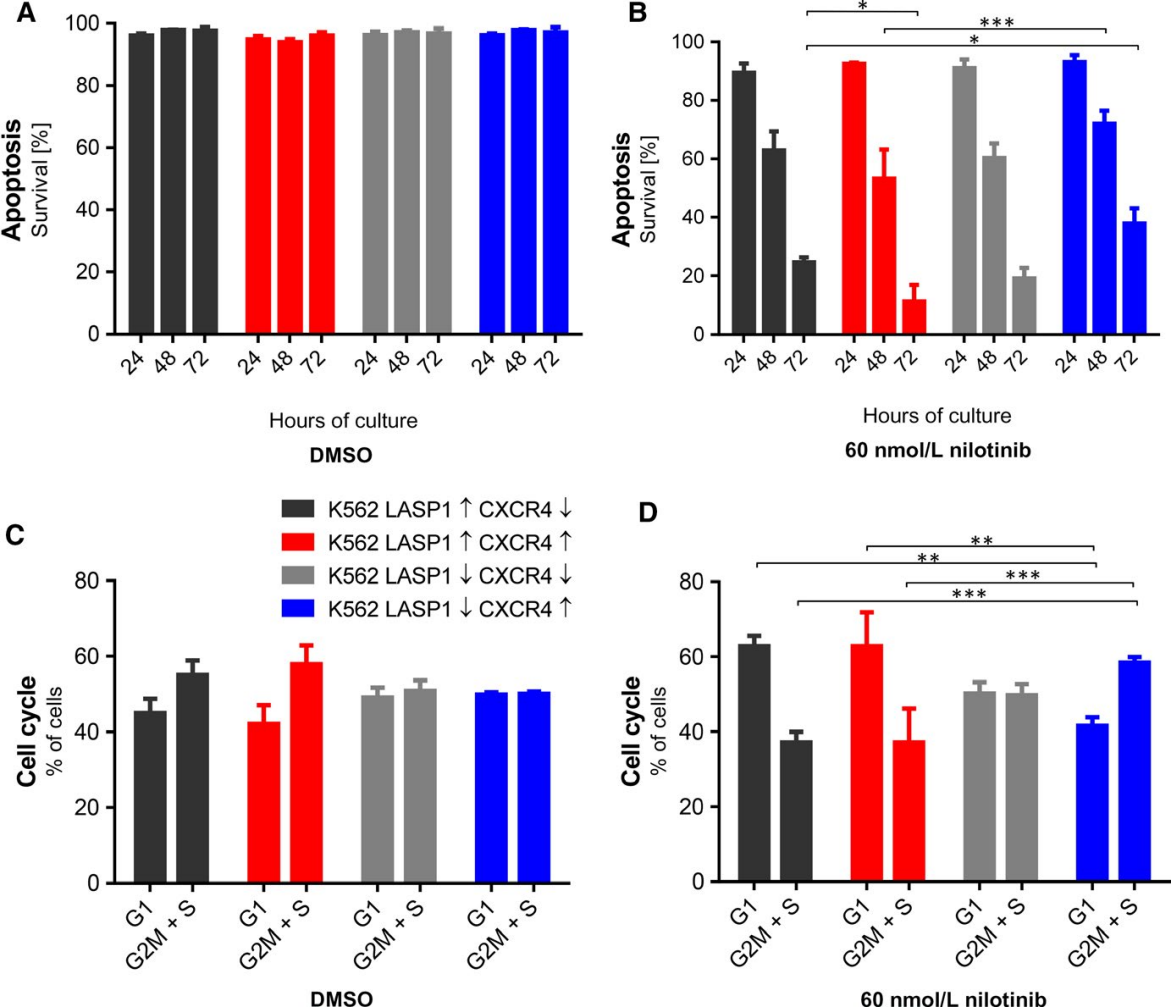
Influence of LASP1 knockout and CXCR4 overexpression in K562 cell lines on apoptosis, cell cycle and flow adhesion. Apoptosis (A and B) and cell cycle arrest (C and D) were measured cytometrically using annexin V/PI staining. LASP1 knockout was of advantage in CXCR4 overexpressing cells while co‐expression of both proteins increased susceptibility towards TKI treatment. Results represent the mean of three independent experiments ± SD. CXCR4, chemokine receptor 4; DMSO, dimethyl sulfoxide; LASP1, LIM and SH3 domain protein 1; PI, propidium iodide; SD, standard deviation; TKI, tyrosine kinase inhibitor

To analyse a possible effect of LASP1 on TKI‐induced cell death in CML, we investigated apoptosis (Figure S2) and cell cycle arrest (Figure S3) in the four generated K562 cell lines using Annexin‐V and propidium iodide (PI) staining, respectively. While there was no major effect on nilotinib‐induced cell death by LASP1 knockout alone, LASP1 depletion in combination with CXCR4 expression significantly impaired nilotinib‐induced apoptosis (Figure 3A,B). In contrast, overexpression of LASP1 and CXCR4 showed increased susceptibility to TKI treatment (comparison of K562‐LASP1↓‐CXCR4↑ and K562‐LASP1↑‐CXCR4↑ with LASP1↑‐CXCR4↓ after 48 and 72 hours for all conditions).

Cell cycle analysis supported these observations. Tyrosine kinase inhibitor treatment significantly increased the percentage of cells in G1 phase and, hence, decreased the proportion of cells detected in G2/M and S phase in K562‐LASP1↑‐CXCR4↓ and K562‐LASP1↑‐CXCR4↑ (Figure 3C,D). In contrast, K562‐LASP1↓‐CXCR4↑ cells showed a slight increase in cells in G2/M phase, while fewer cells were found in G1 phase [comparison of DMSO (Figure 3C) and 60 nmol/L nilotinib (Figure 3D)]. Plerixafor itself had no effect on cell cycle (Figure S3).

Taken together, these results suggest a survival advantage of CML cells overexpressing CXCR4 in the presence of low LASP1 levels.

### 3.5 LASP1 knockout impairs migration but has no significant effect on adhesion of CML cells

**Figure 4:**
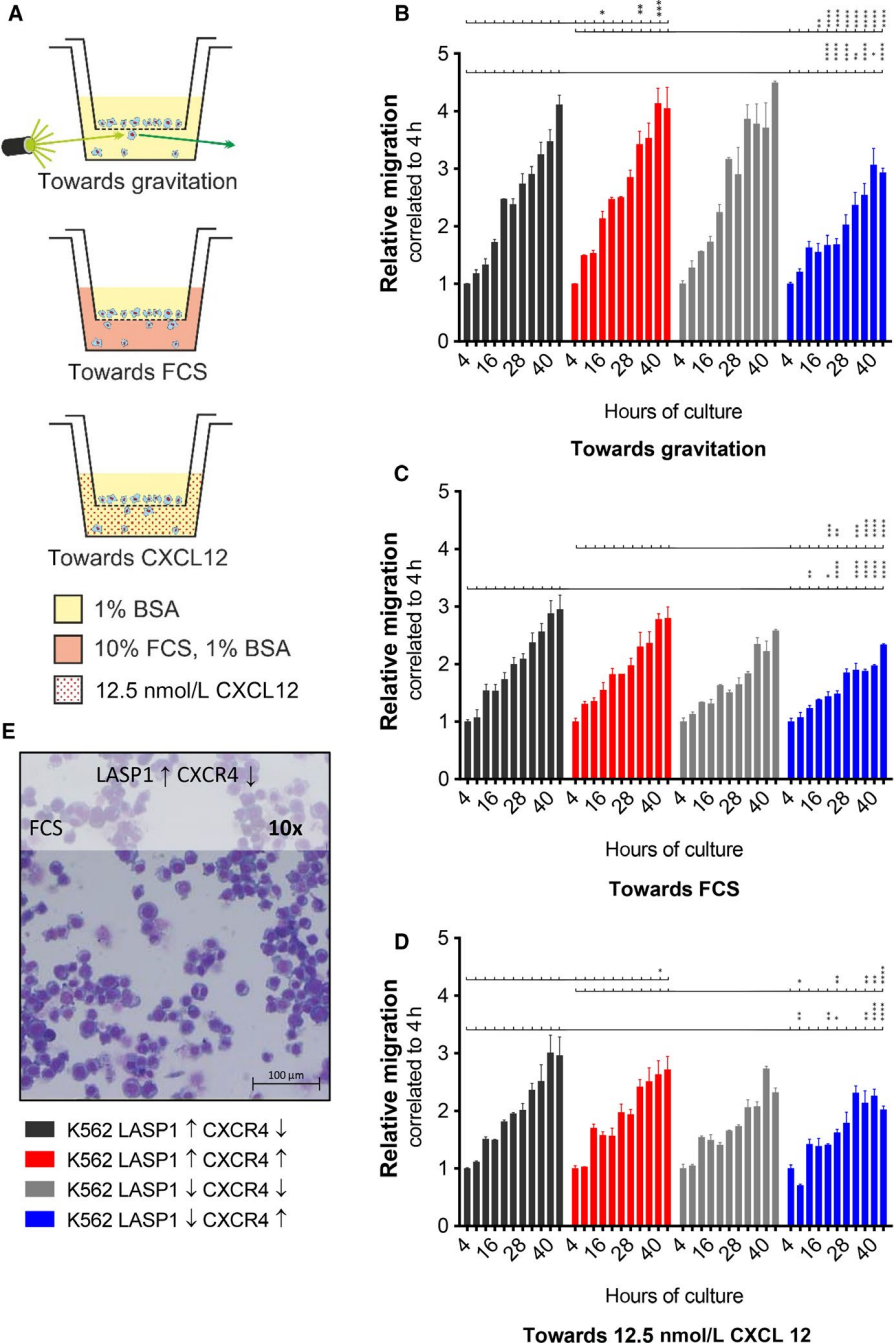
Importance of LASP1 and CXCR4 for migratory potential. (A) Schematic of a migration chamber and assay conditions: Fluorescent signal from actively migrating cells was detected from below without shine‐through artefacts. Relative migration towards gravitation (B), 10% FCS (C) and 12.5 nmol/L (100 ng/mL) CXCL12 (D). In the presence of CXCR4, knockout of LASP1 resulted in reduced migration. (E) Cytospin and Pappenheim staining of migrated cells. Cytospins were prepared as previously described.^9^ Results represent the mean of three independent experiments in duplicates ± SD. BSA, bovine serum albumin; CXCL12, C‐X‐C motif chemokine 12; CXCR4, chemokine receptor 4; FCS, foetal calf serum; LASP1, LIM and SH3 domain protein 1; SD, standard deviation

The importance of CXCR4 for migration and adhesion in CML cells has already been demonstrated.^28^ So far, LASP1 involvement in migratory processes has only been demonstrated for solid tumours.^11^ Therefore, we aimed to characterize the role of CXCR4 and LASP1 in CML cell migration using the different K562 cell lines (Figure S4). A schematic drawing and assay conditions are provided in Figure 4A. The technical setup of the instruments used in this experiment ensured that only active migrating cells are detected on the underside of the membrane. In the presence of CXCR4, LASP1‐depleted cells (K562‐LASP1↓‐CXCR4↑) showed reduced migration in comparison with cells harbouring LASP1 wild‐type (K562‐LASP1↑‐CXCR4↑) under basal conditions (Figure 4B), as well as in the presence of FCS (Figure 4C) and CXCL12 (Figure 4D). Pappenheim staining of cytospin‐fixed migrated cells was used to confirm cell migration visually (Figure 4E).

**Figure 5:**
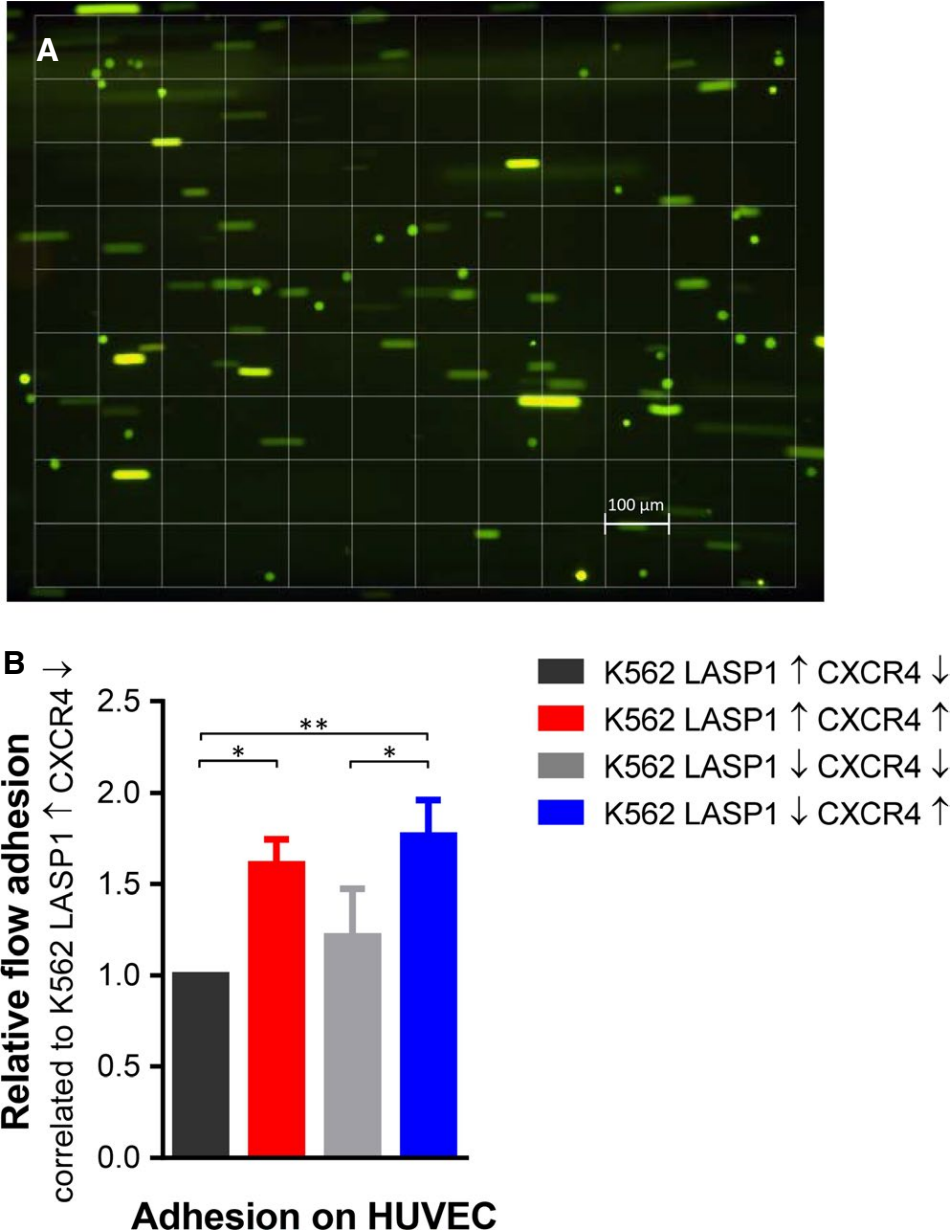
Effect of LASP1 on K562 cell adhesion. A, Cell adhesion was tested under flow conditions. Adherent cells are visible as dots, floating cells as lines. 1 square is equivalent to 1 mm^2^. B, LASP1 knockout reinforced adhesion by trend. Results represent the mean of three independent experiments ± SD. CXCR4, chemokine receptor 4; HUVEC, human umbilical vein endothelial cells; LASP1, LIM and SH3 domain protein 1; SD, standard deviation

Adhesion of the K562‐generated cell lines was tested by counting pre‐labelled K562 cells on a HUVEC monolayer under flow conditions (Figure 5A). In the presence of CXCL12, CXCR4 expression led to a significant increase in adhesion. The process seemed to be augmented by the knockout of LASP1; however, this effect was not statistically significant (Figure 5B).

### 3.6 CXCR4 overexpression and LASP1 knockout attenuate natural killer cell‐mediated cytotoxicity

**Figure 6:**
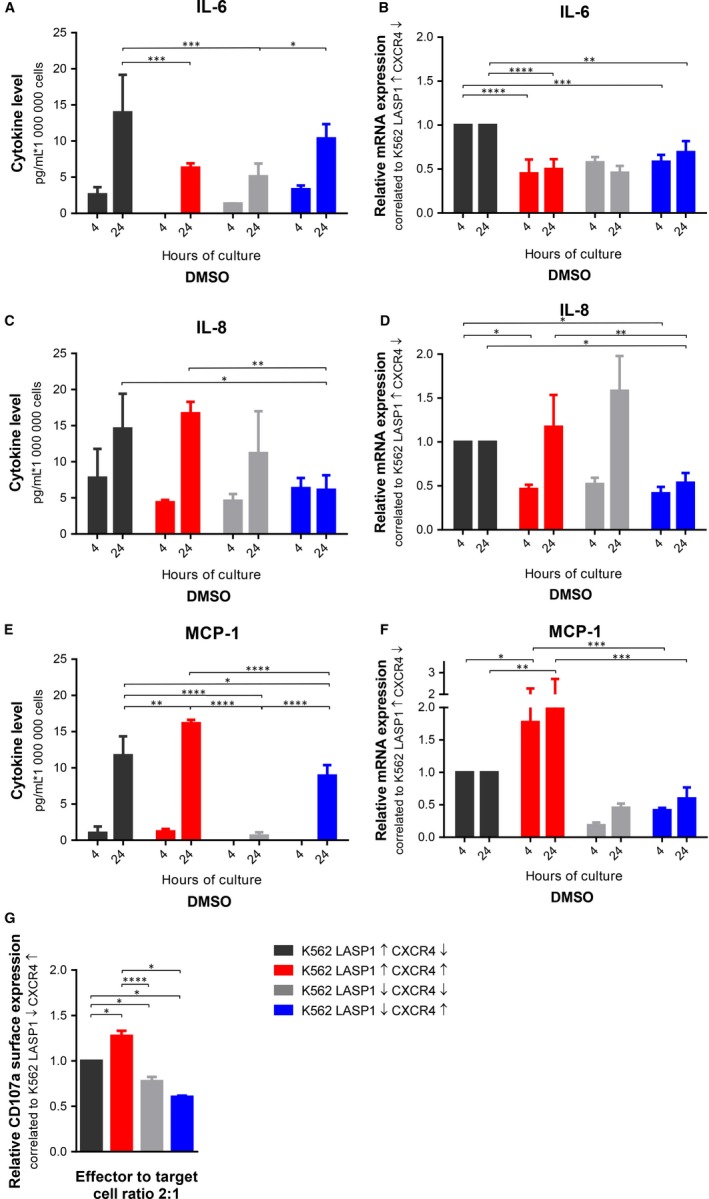
Effect of LASP1 and CXCR4 on K562 cytokine release and NK cell degranulation. Cytokine release of IL‐6 (A), IL‐8 (C) and MCP‐1 (E) into the conditional medium was measured using a bead‐based immunoassay. Relative mRNA expression of IL‐6 (B), IL‐8 (D) and MCP‐1 (F). G, Degranulation response, assessed by flow cytometric analysis of CD107a surface expression of NK‐92C after 4‐h co‐incubation with K562 cells. Results represent the mean of three independent experiments ± SD. CXCR4, chemokine receptor 4; DMSO, dimethyl sulfoxide; LASP1, LIM and SH3 domain protein 1; MCP‐1, monocyte chemoattractant protein‐1; SD, standard deviation

Microarray analyses suggested an impact of LASP1 on immune cell differentiation and function (Table S2). Therefore, we analysed the secretion of inflammatory cytokines into the supernatant of all four cell lines using the bead‐based LEGENDplex™ Human Inflammation Panel immunoassay. In general, nilotinib treatment lowered the secretion of all cytokines, while CXCL12 stimulation showed stable or enhanced cytokine synthesis compared to DMSO controls (Figure S5). Measurement revealed an indifferent cytokine release after CXCR4 depletion under basal conditions, while LASP1 knockout clearly led to a significant decrease of cytokine release (Figure 6A,C,E). As LASP1 is known to affect transcriptional activity 16, 32 and to enhance secretory processes,^32^, ^33^, ^34^ we used qRT‐PCR (Figure 6B,D,F) to differentiate between reduced cytokine release solely due to lowered transcriptional expression or a direct effect of LASP1 on the vesicular secretory process. Our results demonstrate that, despite higher mRNA levels in the LASP1‐knockout cells (Figure 6F), MCP‐1 release is reduced (Figure 6E), while the secretion of IL‐6 and IL‐8 followed their expression levels (Figure 6B,D).

To establish a possible link between CXCR4 and LASP1 expressions, altered cytokine release and evasion of CML cells from the immune system, we tested the susceptibility of the K562 model cell lines to the human natural killer cell line NK‐92C. Effector degranulation was measured as CD107a surface exposure on NK cells by FACS analysis upon target cell recognition as previously described.^23^

As expected, co‐incubation of the generated K562 cells with NK‐92C resulted in a degranulation response. Compared to K562 cells control cells (K562‐LASP1↑‐CXCR4↓), up‐regulation of CXCR4 (K562‐LASP1↑‐CXCR4↑) resulted in increased CD107a surface exposure on NK cells. However, knockout of LASP1 in CXCR4‐overexpressing cells had a contrary effect and significantly lowered the degranulation response of NK cells. This effect is visible in all effector to target cell ratios, while the most severe results were observed for the highest effector to target cell ratio 2:1 (Figure 6G and Figure S7). These findings support our hypothesis that CXCR4 up‐regulation in combination with LASP1 down‐regulation may enable CML cells to evade the innate immune response.

**Figure 7:**
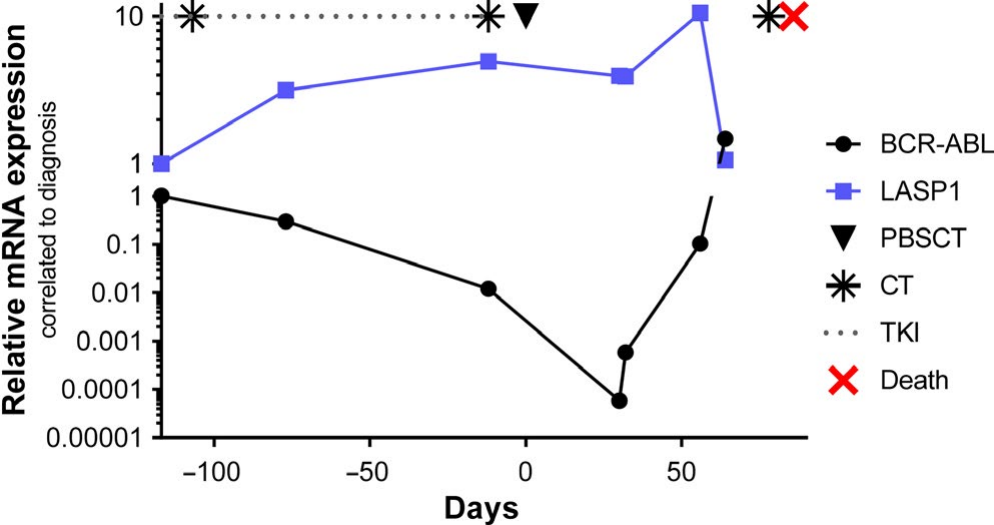
Negative effect of impaired LASP1 levels worsen patients' outcome: a case presentation. A 66‐year‐old woman presented 5 months after being diagnosed to suffer from CML in chronic phase in 2011 (157.4 Gpt/L white blood cells) at the university hospital in lymphatic blast crises with low LASP1 levels (blue line), despite initiated imatinib therapy. Cytarabin, vincristine and hydroxycarbamide (indicated as 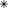) were administered unsuccessfully. As cytogenetics finally revealed the presence of Y253H, E255K and T315I mutations, ponatinib (dotted line) was initiated. Subsequently, the administered pre‐phase chemotherapy consisted of methotrexate, dexamethasone and cyclophosphamide (indicated as 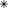) according to German Multicenter Study Group for Adult Acute Lymphoblastic Leukemia.^44^ Induction therapy had to be interrupted due to clinical deterioration. Finally, the patient underwent conditioning therapy with treosulfan, fludarabine and antithymocyte globulin^45^ followed by allogenic peripheral blood stem cell transplantation of 4.1 × 10^6^ CD34+ cells/kg bodyweight (indicated as ▼) from an unrelated male, human leucocyte antigen allele matched 10/10 donor. After peripheral blood stem cell transplantation, BCR‐ABL levels declined, while LASP1 levels increased. Due to a renewed blast crisis (and concomitant lowered LASP1 levels), the patient died 83 d after SCT (indicated as 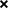) despite further doses of cyclophosphamide (indicated as 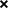). The provenience and preparation of blood samples have been described before.^9^ CML, chronic myeloid leukaemia; CT, chemotherapy; LASP1, LIM and SH3 domain protein 1; PBSCT, peripheral blood stem cell transplantation; TKI, tyrosine kinase inhibitor

The negative effect of impaired LASP1 levels on patient outcome is supported by the case of a 66‐year‐old woman diagnosed to suffer from CML in chronic phase in 2011. Only 5 months later, she was found to have developed a blast crisis with evidence of Y253H, E255K and T315I mutations. Relative LASP1 expression levels inversely mirrored those of BCR‐ABL during her treatment (Figure 7).

In addition, 57 patients newly diagnosed to suffer from CML in chronic phase were analysed. Median LASP1 mRNA levels correlated with βGUS mRNA were 7.383 (range 2.965‐39.518) in the patient cohort compared to the control group's median of 7.492 (range 6.245‐9.825; five healthy participants; *P* = .7827). These preliminary data suggest a wider distribution of LASP1 mRNA expression levels in CML patients. However, further investigations are needed to validate these findings.

## 4 DISCUSSION

Over the past years, increasing knowledge about the interactions between leukaemic cells and the BM has provided insights into mechanisms of cell survival and CML resistance towards therapeutic agents. Although BCR‐ABL expressing cells have functional defects in the CXCR4 signalling axis, imatinib treatment increases CXCR4 expression, and CXCL12 activation improves BM homing and survival of CML stem cells.^8^, ^35^ Based on recently published data demonstrating a binding of LASP1 and CXCR4 in breast cancer cells, we investigated the role of LASP1 in the light of this interaction for CML.^15^ To do so, we generated cell lines with inactivated LASP1 and/or CXCR4 overexpression using a CRISPR/Cas9‐based LASP1 knockout system and a lentivirus‐mediated CXCR4 overexpression in K562 cells.

Our data indicate that down‐regulation of LASP1 promotes survival advantages for CML cells, especially when treated with TKI. Data above demonstrate that abrogation of *LASP1* expression levels promotes resistance towards TKI treatment, reduces migration, increases adhesive behaviour and contributes to impaired recognition by the immune system. Thereby, our results provide the first cell‐based confirmation of the bioinformatics data by Yeung and colleagues, who predicted that a reduced LASP1 concentration might be unfavourable during CML progression, as they observed lower LASP1 mRNA levels in blast crisis patients.^13^

Looking at the bigger picture of LASP1 involvement in malignancy, our results are in contrast to data on solid tumours, in which LASP1 overexpression contributes to cancer aggressiveness,^10^ thus implementing major biochemical differences of LASP1 action in united cell structures and haematological cells.

In certain solid tumours, hypoxic conditions lead to an up‐regulation of LASP1^12^ by binding of HIF1α to a hypoxia response element in the *LASP1* promotor region.^25^ Although the environment in the BM was shown to be hypoxic,^27^ we were not able to identify a positive correlation between LASP1 and HIF1α in CML within the microarray data sets.

In solid tumours, localization of LASP1 is not restricted to the cytoplasm, as the protein can also be found within the nucleus. Nuclear localization significantly correlates with poor outcome in breast cancer^36^ and hepatocellular carcinoma.^37^ Mechanistically, phosphorylation of LASP1 at S146 allows an interaction with CXCR4.^15^ Activation by CXCL12 results in the release of LASP1 from the receptor, subsequent translocation into the nucleus through the interaction with zona occludens 2 (ZO‐2)^12^ and binding to nuclear Snail, ubiquitin‐like with PHD and ring finger domains 1 (UHRF1) and histone methyltransferase G9a.^15^ We have not been able to detect LASP1 within the nucleus of CML cells, yet,^9^ most likely because these cells harbour no ZO‐2.^9^ Expression and activation of CXCR4 in K562 cells had no impact on ZO‐2 levels (data not shown). Similarly, expression of known LASP1 binding partners like β‐actin, VASP, dynamin, vimentin, FAK or CRKL, involved in cytoskeleton or adhesion complexes,^12^ was not affected by LASP1 knockout or CXCR4 up‐regulation (Figure S8). Solely, zyxin showed a 50% up‐regulation in LASP1‐depleted cells compared to LASP1 expressing cells. This compensatory effect might explain the observed augmented, albeit not significant, adhesion of K562 cells on HUVEC monolayers under flow conditions (Figure 5B; K562‐LASP1↑‐CXCR4↓ vs K562‐LASP1↓‐CXCR4↓ and K562‐LASP1↑‐CXCR4↑ vs K562‐LASP1↓‐CXCR4↑) as zyxin is known to concentrate at focal adhesions.^38^ The important and well‐known effect of CXCR4 on adhesion is not due to zyxin expression as CXCR4 up‐regulation is not affecting zyxin levels (Figure S8).

Cytokine release plays an important role in the regulation of the immune response. Our data revealed an influence of LASP1 on MCP‐1 release from target cells. While CXCR4 expressing cells showed enhanced MCP‐1 secretion, LASP1 depletion in K562 cells resulted in decreased MCP‐1 levels in the conditional medium. An effect of LASP1 on secretory processes has already been observed for secretion of hydrochloric acid in gastric parietal cells, melanin budding from epidermal melanocytes, matrix metalloprotease release from breast cancer cells^32^, ^33^, ^34^ and a direct LASP1 influence on F‐actin‐dynamin vesicle budding is discussed^33^, ^34^—a process also facilitating cytokine release.^39^

MCP‐1 promotes T‐lymphocyte differentiation, serves as a chemoattractant and even activates NK cells.^40^ As lower LASP1 levels lead to a reduced K562 cytokine release and reduced CD107a surface expression on NK cells, lower LASP1 levels in CML patients might contribute to the evasion of CML cells from the immune system, especially since effector NK cells are per se reduced in number and have a limited cytolytic capacity in CML.^41^ TKI treatment further impairs this cellular immune function.^41^

The results from this study clearly demonstrate a role for LASP1 in CML progression. Knockout of LASP1, as a model for the observed reduced LASP1 levels in non‐responders and blast crisis patients, enhances proliferation under TKI treatment, impairs migration and favours survival of CML cells. Furthermore, LASP1‐depleted cells show reduced cytokine release and allow CML cells to evade NK cell degradation. Thus, our data point to an involvement of CXCR4 itself in adhesion and NK cell‐mediated cytotoxicity. Finally, the combination of CXCR4 overexpression and LASP1 down‐regulation seems to promote resistance towards TKI‐mediated apoptosis and appears to be advantageous for sustained proliferation. LASP1 might therefore constitute an additional new prognostic marker to identify CML patients in risk of relapse.

Our data not only indicate a role of LASP1 for CML progression, but, with respect to the microarray data, also suggest an impact of the protein in leukaemic stem cell behaviour.^28^, ^42^ Even though the K562 cell line is not really suited for drawing conclusions regarding leukaemic stem cells, some findings are similar to cell behaviour observed in LSCs: Stem cell quiescence is closely related to apoptotic resistance as these cells are not fully eliminated by cell cycle‐specific drugs or TKIs.^26^, ^43^ Promoting cell cycle re‐entry of quiescent leukaemic stem cells results in better outcome of CML treatment, but also affects long‐term capacity for survival and self‐renewal of hematopoietic stem cells.^26^ Our data from the K562 model shows that despite nilotinib treatment, knockout of LASP1 results in higher amount of cells entering G2 phase (Figure 3D). In cells overexpressing CXCR4, LASP1 depletion even shows improved survival rates (Figure 3B). It is therefore possible that TKI resistance in LASP1 depleted K562 and drug resistance of quiescent LSCs with low LASP1 expression share the same mechanism. In addition, a tendency of LASP1‐depleted K562 cells to increased adhesion is observed (Figure 5B). Whether this contributes to cell adhesion‐mediated drug resistance (CAM‐DR) has to be further elucidated.^26^

In order to properly investigate LASP1 effects in LSCs, further work has to be done including ex vivo studies of sorted patient stem cells and animal models.

## CONFLICT OF INTEREST

EB and JF have received grants from the German Cancer Aid (Project numbers: 70112717 and 70112142). The authors declare that they have no conflicts of interests. Content has not been published in similar or the same form elsewhere.

## AUTHOR CONTRIBUTIONS

All authors read and approved the final manuscript. EB and JJF conceived and planned the experiments. MFO analysed the microarray data, EB cloned the CXCR4 receptor, and JPM carried out lentiviral transfection. ABH, MLM and JJF carried out experiments. ABH, EB and JJF analysed and compiled the data and wrote the manuscript.

## ETHICAL APPROVAL

The study has been approved by the institutional ethics committee, and patients provided written informed consent in accordance with the Declaration of Helsinki.

## Data Availability

The data that support the findings of this study are available from the corresponding author upon reasonable request.
