# Supplementary material for: Knockout of LASP1 in CXCR4 expressing CML cells promotes cell persistence, proliferation and TKI resistance
Source: J Cell Mol Med. 2020 Jan 19;24(5):2942–55. doi: 10.1111/jcmm.14910 (PMC7077607; doi:10.1111/jcmm.14910)
Supplement: Supplementary file 1 [file JCMM-24-2942-s001.pdf]

Supplemental Material

Knockout of LASP1 in CXCR4 expressing CML cells promotes cell persistence, proliferation and TKI resistance

Supplemental Materials and Methods

Cell culture conditions

K562 cells were cultured in RPMI + GlutaMAX-1 (Gibco™, ThermoFisher, Dreieich, Germany) supplemented with 10% FCS (Biochrom, Berlin, Germany). Human umbilical vein endothelial cells (HUVEC) were cultured in HUVEC Mix Medium consisting of one part Endothelial Cell Growth Medium (C-22220; PromoCell, Heidelberg, Germany) supplemented with Growth Medium MV SupplementPack (C-39220, PromoCell) and two parts M199 medium (Gibco™) supplemented with 8.4% FCS, 63 nM gentamycin and 0.18 IU/ml heparin (Sigma-Aldrich, Taufkirchen, Germany). Natural killer cell line 92C (NK-92C) was cultured in Alpha-MEM supplemented with 10% horse serum (Sigma-Aldrich) and 10% FCS. The K562 cell line was purchased from ATCC (Manassas, VA, USA) and HUVEC from PromoCell (Heidelberg, Germany). NK-92C cells were a kind gift from Prof. Dr. rer. nat. Carsten Watzl, Leibniz Research Centre for Working Environment and Human Factors (IfADo, TU-Dortmund, Dortmund, Germany). All cell lines were cultured under 5% CO_2 at 37°C in a humidified atmosphere and tested for mycoplasma contamination.

Drug formulations

BCR-ABL activity was blocked using nilotinib, and CXCR4 was inhibited by plerixafor at concentrations indicated (Cayman Chemical, Ann Arbor, MI, USA). Nilotinib was dissolved to a 10 mM (5.3 mg/ml) stock solution in dimethyl sulfoxide (DMSO; Sigma-Aldrich).

Plerixafor was diluted in PBS to a final concentration of 10 mM just before administration. C-X-C motif chemokine 12 (CXCL12) and tumor necrosis factor alpha (TNF-α) were purchased from INVIGATE GmbH (Jena, Germany) and diluted in PBS containing 0.1% BSA to a concentration of 12.5 µM (100 µg/ml) and 286 nM (5 µg/ml), respectively. Nilotinib, CXCL12 and TNF-α stock solutions were stored at -20°C. Stock solutions were then diluted to the final concentration as indicated.

Generation of LASP1 knockout cells

For the generation of LASP1 knockout cells, a mixture of 2.0 µg DNA/2.5 × 10^5 cells/well, 7.5 µg UltraCruz® Transfection Reagent and 1 ml Plasmid Transfection Medium was added and incubated for 24 h. After selection with 0.8 µg/ml puromycin (Santa Cruz, Heidelberg, Germany) for 48 h, cells were additionally sorted for high GFP positivity using BD FACSAria™ and BD FACSDiva™ software (Becton Dickinson, Heidelberg, Germany). The transfection rate ranged between 10 - 20%.

Cloning of CXCR4

Human CXCR4 was amplified by PCR from human peripheral blood leukocyte cDNA, using the following primers: 5’-GCT CTA GA-G CCA CCA TGG AGG GGA TCA GTA TAT ACA CTT CAG AT-3’ and 5’-GGA ATT CTT AGC TGG AGT GAA AAC TTG AAG A-3’. The PCR products were cloned into the pCDH-CMV-MCS-EF1-Puro plasmid (Addgene Europe, Teddington, UK) using Xba1 and EcoR1 restriction enzymes (New England BioLabs GmbH, Frankfurt, Germany). Correct CXCR4 sequences were confirmed by sequencing (Genetic Analyzer 3500, Applied Biosystems, Germany).

Western blot analysis

Western blot analysis was carried out as described before [1]. The polyclonal rabbit LASP1 antibody has been described previously [2]. Antibodies against CXCR4 (ab124824) and VASP (ab 229624) were purchased from abcam (Berlin, Germany). For CXCR4 Western blots, samples were not boiled. Antibodies against AKT1 (#9271), phospho-AKT1-S473 (#9275), phospho-AKT1-T308 (#9271), Vimentin (#5741) and ZO-2 (#2847) were purchased from Cell Signaling (Frankfurt, Germany). FAK (sc-271126), CRKL (sc-319) and Dynamin (sc-17807) antibodies were purchased from Santa Cruz. Zyxin (#307011) was purchased from SySy (Göttingen, Germany). Equal protein loading was confirmed by analyzing β-actin level using the goat polyclonal anti-β-actin antibody (sc-1616; Santa Cruz).

RT-PCR analysis

Total RNA was extracted from cells using the innuPREP RNA Mini Kit 2.0 (Analytik Jena, Jena, Germany) according to manufacturer's instructions. First-strand cDNA was generated from 1 µg of total RNA using M-MLV reverse transcriptase according to manufacturer's protocol (Invitrogen, Karlsruhe, Germany). BCR-ABL, β-glucuronidase (GUSB), CXCR4, IL-6, IL-8, LASP1, LASP2, and MCP-1 mRNA levels were evaluated with SYBR Green using the Mastercycler® ep realplex Real-time PCR System (Eppendorf, Hamburg, Germany). Primers were designed to meet specific criteria by using the Primer3 software (http://frodo.wi.mit.edu). Primers for detection of GUSB and BCR-ABL were used as described before [3,4]. Primers were designed to over-span introns, except LASP1-h2-1 and LASP1-h2-2, which were used to check for successful down regulation (Supplemental Table 1). The reaction set up (20 µL) was as follows: 20 ng cDNA, 0.5 µM of each primer and 1 × FastStart SYBR Green Master (Roche, Mannheim, Germany). All reactions were carried out at 95°C for 10 min followed by 45 cycles at 95°C for 10 s, 15 s at the indicated annealing

temperature, and 72°C for 25 s. Melting curve analyses were performed. Changes in expression levels were normalized to β-glucuronidase (GUSB) of K562-LASP1↑-CXCR4↓ treated with DMSO using the ΔΔCt method of relative quantification [5].

Sanger sequencing

gDNA was obtained using the QIAamp DNA Mini Kit (Qiagen, Hilden, Germany) according to the manufacturer's recommendations. The sequencing reaction mix was prepared by using the BigDye Terminator Cycle Sequencing Kit v3.1 (Life Technologies, Darmstadt, Germany) according to the manufacturer's instructions. Sanger sequencing of PCR products covering the relevant genetic regions (sequence forward primer: AGA CAC TAC CCC AAG CAG TC; reverse primer ATT TCA GCA CCT TGG CCT T) was performed as described before [6] using a ABI3500 Genetic Analyzer (Life Technologies).

Apoptosis assay

Cells were incubated with compounds as indicated. The cells were washed with PBS and resuspended in 200 µL staining solution containing 0.25 µL Annexin-V-allophycocyanin (APC), 0.5 µL propidium iodide (PI) and 199.25 µL Annexin-V binding buffer (all BD Pharmingen™, Heidelberg, Germany) for 15 min in the dark at RT. After addition of 400 µL Annexin-V binding buffer per sample, the cells were analyzed by flow cytometry using a BD FACSCalibur™ (Becton Dickinson) [7].

Cell cycle analysis

Cells were washed twice with cold PBS and fixated at −20°C for 30 min with 70% (v/v) ethanol. Cells were then pelleted and washed with cold PBS and analyzed using PI staining. In brief, cells were incubated for 30 min with a 2 mg/100 ml PI (BD Pharmingen™) solution

containing 20 mg/100 ml RNase A (ThermoFisher) and 0.1% (v/v) Triton X (Sigma-Aldrich) [8]. Samples were analyzed on a BD FACSCalibur™ using the BD CellQuest™ (Becton Dickinson) and FlowJo® software (Tree Star, USA).

Cell viability analysis

Cell viability assays were performed using the RealTime-Glo™ MT Cell Viability Assay according to the manufacturer's protocol (Promega, Madison, WI, USA). Cells were seeded at a density of 5×10^3 cells/well in triplicates into a black-walled 96-well plate and incubated with the compounds as indicated. For time-zero measurements, cells were incubated with RealTime-Glo™ MT Cell Viability reagent for 1 h at 37°C, and luminescence was measured on a CLARIOstar microplate reader (BMG Labtech GmbH, Ortenberg, Germany). Luminescence was then measured at 24 h, 48 h, and 72 h after the addition of RealTime-Glo™ reagent.

Adhesion assay

Adhesion assays under flow conditions were performed as previously described [9]. In brief, 1.1×10^5 HUVEC cells were seeded on µ-Slide I 0.4 mm Luer (Ibidi, Munich, Germany) and were allowed to attach for 4 h. HUVEC cells were then stimulated for 16 h with HUVEC Mix Medium supplemented with 286 pM TNF-α. After attachment, the µ-Slide was connected to the pump system containing 5 ml of HUVEC Mix Medium supplemented with 25 nM (200 ng/ml) CXCL12. Simultaneously, 3×10^6 cells were washed and re-suspended in modified HBSS buffer (pH 7.2; 0.5% BSA; 10 mM Hepes; Gibco™). Subsequently, cells were stained with 10 µM carboxyfluorescein succinimidyl ester (CFSE; abcam) for 15 min. Just before introducing the cells to the pump system, Ca^{2+} and Mg^{2+} were added at a final concentration of 1 mM. Cells were immediately set under flow for 10 min with a flow rate of

0.25 ml/min and a shear stress of 0.33 dyn/cm^2. Quantification was done by microscopically counting adherent cells in 10 adjacent spots of 1 mm^2 along the perfusion channel (Leica DM4000 B LED; Wetzlar, Germany; Ex: 492 nm; Em: 517 nm; 10x magnification, see Fig. 3E) and DISKUS software (Carl H. Hilgers, Königswinter, Germany).

Supplemental References

1. **Frietsch JJ, Kastner C, Grunewald TG, Schweigel H, Nollau P, Ziermann J, Clement JH, La Rosee P, Hochhaus A, Butt E.** LASP1 is a novel BCR-ABL substrate and a phosphorylation-dependent binding partner of CRKL in chronic myeloid leukemia. *Oncotarget*. 2014; 5: 5257-71.
2. **Butt E, Gambaryan S, Gottfert N, Galler A, Marcus K, Meyer HE.** Actin binding of human LIM and SH3 protein is regulated by cGMP- and cAMP-dependent protein kinase phosphorylation on serine 146. *J Biol Chem*. 2003; 278: 15601-7.
3. **Muller MC, Erben P, Saglio G, Gottardi E, Nyvold CG, Schenk T, Ernst T, Lauber S, Kruth J, Hehlmann R, Hochhaus A, European L.** Harmonization of BCR-ABL mRNA quantification using a uniform multifunctional control plasmid in 37 international laboratories. *Leukemia*. 2008; 22: 96-102.
4. **Emig M, Saussele S, Wittor H, Weisser A, Reiter A, Willer A, Berger U, Hehlmann R, Cross NC, Hochhaus A.** Accurate and rapid analysis of residual disease in patients with CML using specific fluorescent hybridization probes for real time quantitative RT-PCR. *Leukemia*. 1999; 13: 1825-32.
5. **Livak KJ, Schmittgen TD.** Analysis of relative gene expression data using real-time quantitative PCR and the 2(-Delta Delta C(T)) Method. *Methods*. 2001; 25: 402-8.
6. **Rinke J, Schafer V, Schmidt M, Ziermann J, Kohlmann A, Hochhaus A, Ernst T.** Genotyping of 25 leukemia-associated genes in a single work flow by next-generation sequencing technology with low amounts of input template DNA. *Clin Chem*. 2013; 59: 1238-50.
7. **Zirm E, Spies-Weisshart B, Heidel F, Schnetzke U, Bohmer FD, Hochhaus A, Fischer T, Scholl S.** Ponatinib may overcome resistance of FLT3-ITD harbouring additional point mutations, notably the previously refractory F691I mutation. *Br J Haematol*. 2012; 157: 483-92.
8. **Darzynkiewicz Z, Juan G, Bedner E.** Determining cell cycle stages by flow cytometry. *Curr Protoc Cell Biol*. 2001; Chapter 8: Unit 8 4.
9. **Ende G, Poitz DM, Wiedemann E, Augstein A, Friedrichs J, Giebe S, Weinert S, Werner C, Strasser RH, Jellinghaus S.** TNF-alpha-mediated adhesion of monocytes to endothelial cells-The role of ephrinA1. *J Mol Cell Cardiol*. 2014; 77: 125-35.
10. **McWeeney SK, Pemberton LC, Loriaux MM, Vartanian K, Willis SG, Yochum G, Wilmot B, Turpaz Y, Pillai R, Druker BJ, Snead JL, MacPartlin M, O'Brien SG, Melo JV, Lange T, Harrington CA, Deininger MW.** A gene expression signature of CD34+ cells to predict major cytogenetic response in chronic-phase chronic myeloid leukemia patients treated with imatinib. *Blood*. 2010; 115: 315-25.
11. **Irizarry RA, Hobbs B, Collin F, Beazer-Barclay YD, Antonellis KJ, Scherf U, Speed TP.** Exploration, normalization, and summaries of high density oligonucleotide array probe level data. *Biostatistics*. 2003; 4: 249-64.
12. **Dai M, Wang P, Boyd AD, Kostov G, Athey B, Jones EG, Bunney WE, Myers RM, Speed TP, Akil H, Watson SJ, Meng F.** Evolving gene/transcript definitions significantly alter the interpretation of GeneChip data. *Nucleic Acids Res*. 2005; 33: e175.
13. **Subramanian A, Tamayo P, Mootha VK, Mukherjee S, Ebert BL, Gillette MA, Paulovich A, Pomeroy SL, Golub TR, Lander ES, Mesirov JP.** Gene set enrichment analysis: a knowledge-based approach for interpreting genome-wide expression profiles. *Proc Natl Acad Sci U S A*. 2005; 102: 15545-50.

Supplemental Table Legends

Supplemental Table 1: Primer Sequences

Primers used for amplification quantification of mRNA, corresponding annealing temperature. CXCR4: chemokine receptor 4; LASP1: LIM and SH3 domain protein 1; MCP-1: monocyte chemoattractant protein-1

Supplemental Table 2: GSEA: Top 50 results from GSEA for LASP1 co- and antiregulated gene sets in imatinib non-responders and responders

Publicly available microarray data sets of isolated CD34-positive BM cells were discriminated in TKI-responders and non-responders [10]. The according CEL files were normalized with RMA [11] using a custom brainarray CDF [12]. A pre-ranked GSEA was performed [13] utilizing curated gene sets from the Broad Institute, NES and p-value corrected for FWER and FDR. BM: bone marrow; FDR: false discovery rate; FWER: family-wise error rate; GSEA: gene set enrichment analysis; NES: Normalized Enrichment Score; TKI: tyrosine kinase inhibitor.

Supplemental Figure legends

Supplemental Figure S1: Effect of LASP1 knockout and CXCR4 overexpression on cell viability

K562-LASP1↑-CXCR4↑, K562-LASP1↑-CXCR4↓, K562-LASP1↓-CXCR4↑ and K562-LASP1↓-CXCR4↓ cell lines were tested for viability after indicated time points. Cells were incubated with (A) DMSO (control), (B) 30 nM nilotinib, (C) 60 nM nilotinib, (D) 120 nM nilotinib, (E) 5 µM and (F) 12.5 nM CXCL12, (G) simultaneous incubation with 12.5 nM CXCL12 and 120 nM nilotinib, (H) in the presence of 12.5 nM CXCL12 after 1 h preincubation with plerixafor and (I) in the presence of 12.5 nM CXCL12 and 60 nM nilotinib after 1 h preincubation with plerixafor. Simultaneous knockout of LASP1 and up-regulation of CXCR4 results in a reduced sensitivity against nilotinib [best seen with 60 nM nilotinib (C)]. When treated with CXCL12, CXCR4-expressing cells show higher proliferation rates than non-CXCR4 expressing cells. This effect is most obvious after 48 h. After 72 h, all cells are dying. When stimulated with CXCL12, both, CXCR4 expression (K562-LASP1↓-CXCR4↑) and LASP1 expression (K562-LASP1↑-CXCR4↓) showed small advantage in cell growth compared to K562-LASP1↓-CXCR4↓ cells. However, co-expression of LASP1 and CXCR4 (K562-LASP1↑-CXCR4↑) significantly enhanced cell proliferation. The simultaneous incubation of the cells with TK inhibitor and CXCL12 did not restore viability. Likewise, CXCR4 activation had no rescue effect on nilotinib-treated cells; inhibition of CXCR4 by plerixafor had neither. Results represent the mean of three independent experiments in triplicates ± SD. CXCR4: chemokine receptor 4; DMSO: dimethyl sulfoxide; LASP1: LIM and SH3 domain protein 1; SD: standard deviation; CXCL12: C-X-C motif chemokine 12.

Supplemental Figure S2: Influence of LASP1 knockout and CXCR4 overexpression in K562 cell lines on apoptosis

Apoptosis was measured cytometrically using Annexin V/PI staining after incubation with (A) DMSO (control), (B) 30 nM nilotinib, (C) 60 nM nilotinib, (D) 120 nM nilotinib, (E) 12.5 nM CXCL12, and (F) simultaneous incubation with 12.5 nM CXCL12 and 60 nM nilotinib, (G) in the presence of 12.5 nM CXCL12 after 1 h preincubation with plerixafor and (H) in the presence of 12.5 nM CXCL12 and 60 nM nilotinib after 1 h preincubation with plerixafor. Results represent the mean of three independent experiments ± SD. CXCR4: chemokine receptor 4; DMSO: dimethyl sulfoxide; LASP1: LIM and SH3 domain protein 1; PI: propidium iodide; SD: standard deviation; CXCL12: C-X-C motif chemokine 12.

Supplemental Figure S3: Influence of LASP1 knockout and CXCR4 overexpression in K562 cell lines on cell cycle

Cell cycle arrest was measured cytometrically using PI staining after incubation with (A) DMSO (control), (B) 30 nM nilotinib, (C) 60 nM nilotinib, (D) 120 nM nilotinib, (E) after 1 h preincubation with plerixafor, and (F) in the presence of 60 nM nilotinib after 1 h preincubation with plerixafor. Knockout of LASP1 trends to an elevated amount of cells in G1 phase, while TKI treatment significantly decreases the cells in G1 phase. Pretreatment with plerixafor does not fundamentally change cell cycle analysis. Results represent the mean of three independent experiments ± SD. CXCR4: chemokine receptor 4; DMSO: dimethyl sulfoxide; LASP1: LIM and SH3 domain protein 1; SD: standard deviation; CXCL12: C-X-C motif chemokine 12; TKI: tyrosine kinase inhibitor.

Supplemental Figure S4: Importance of LASP1 and CXCR4 for migratory potential

For a schematic of a migration chamber and assay conditions, see Fig. 4. Relative migration towards gravitation (A), FCS (B), FCS in the presence of 60 nM nilotinb (C), 12.5 nM CXCL12 (D), 12.5 nM CXCL12 in the presence of 60 nM nilotinib (E), 12.5 nM CXCL12 after 1 h preincubation with plerixafor (F) and 12.5 nM CXCL12 in presence of 60 nM nilotinib after 1 h preincubation with Plerixafor (G). There was no difference when migrating towards CXCL12 or FCS. Migration was best under serum free conditions and pretreatment with plerixafor did further reduce migration. Results represent the mean of three independent experiments in duplicates ± SD. CXCR4: chemokine receptor 4; FCS: fetal calf serum; LASP1: LIM and SH3 domain protein 1; SD: standard deviation; CXCL12: C-X-C motif chemokine 12.

Supplemental Figure S5: Positive role of LASP1 and CXCR4 for cytokine release

Cytokine release of IL-6, IL-8 and MCP-1 was measured using a bead-based immunoassay under DMSO (A, D, G), in the presence of 60 nM nilotinib (B, E, H) and in presence of 6.75 nM CXCL12 (C, F, I), respectively. The most significant results were obtained for MCP-1. Treatment with nilotinib and knockout of LASP1 resulted in decreased cytokine levels, whereas CXCL12 stimulation revealed increased cytokine levels. Results represent the mean of three independent experiments ± SD. CXCR4: chemokine receptor 4; DMSO: dimethyl sulfoxide; LASP1: LIM and SH3 domain protein 1; MCP-1: monocyte chemoattractant protein-1; SD: standard deviation; CXCL12: C-X-C motif chemokine 12.

Supplemental Figure S6: Positive role of LASP1 and CXCR4 for cytokine expression

Relative expression of IL-6 mRNA (A), IL-8 mRNA (D) and MCP-1 mRNA (G) was analyzed using qRT-PCR as described in the Supplemental Materials and Methods section. Ex-

pression was also analyzed in presence of 60 nM nilotinib (B, E, H, respectively) and 6.75 nM CXCL12 (C, F, I, respectively). Relative expression of CXCR4 mRNA under DMSO (K), 60 nM nilobinib (L) and 6.75 nM CXCL12 (M) revealing successful overexpression of CXCR4. Relative expression of LASP1 (N and O) showing decreased mRNA levels after knockout. Results represent the mean of three independent experiments ± SD. CXCR4: chemokine receptor 4; DMSO: dimethyl sulfoxide; LASP1: LIM and SH3 domain protein 1; MCP-1: monocyte chemoattractant protein-1; SD: standard deviation; CXCL12: C-X-C motif chemokine 12.

Supplemental Figure S7: <u>Positive role of LASP1 and CXCR4 for NK cell degranulation</u>
Degranulation response of NK-92C after 4h co-incubation with K562 cells at indicated ratios was assessed by flow cytometric analysis of CD107a surface expression. While overexpression of CXCR4 in K562 cells expressing normal LASP1 levels results in an increased NK cell degranulation (compare black bars with red bars), downregulation of LASP1 leads to decreased NK cell degranulation (compare red bars with blue bars) Results represent the mean of three independent experiments ± SD. CXCR4: chemokine receptor 4; LASP1: LIM and SH3 domain protein 1; MCP-1: monocyte chemoattractant protein-1; SD standard deviation.

Supplemental Figure S8: <u>Influence of LASP1 knockout and CXCR4 overexpression in K562 cell lines on the expression of selected cytoskeleton proteins</u>
Known LASP1 binding proteins were analyzed by Western Blot for transcriptional effects of LASP1 knockout in the presence or absence of CXCR4. CRKL: CRK-like protein FAK: focal adhesion kinase; LASP1: LIM and SH3 domain protein 1; VASP: vasodilator-stimulated phosphoprotein.

Supplemental Figure S9: Illustration of LASP1 Double Nicking by CRISPR/Cas9

Sanger sequencing of gDNA of K562-LASP1↑-CXCR4↑, K562-LASP1↑-CXCR4↓, K562-LASP1↓-CXCR4↑ and K562-LASP1↓-CXCR4↓ cell lines revealed deletion of 32 base pairs (framed in red) in K562-LASP1↓-CXCR4↑ and K562-LASP1↓-CXCR4↓ cell lines.
