# Supplementary material for: Knockout of LASP1 in CXCR4 expressing CML cells promotes cell persistence, proliferation and TKI resistance
Source: J Cell Mol Med. 2020 Jan 19;24(5):2942–55. doi: 10.1111/jcmm.14910 (PMC7077607; doi:10.1111/jcmm.14910)

Supplemental Figure S1: Viability

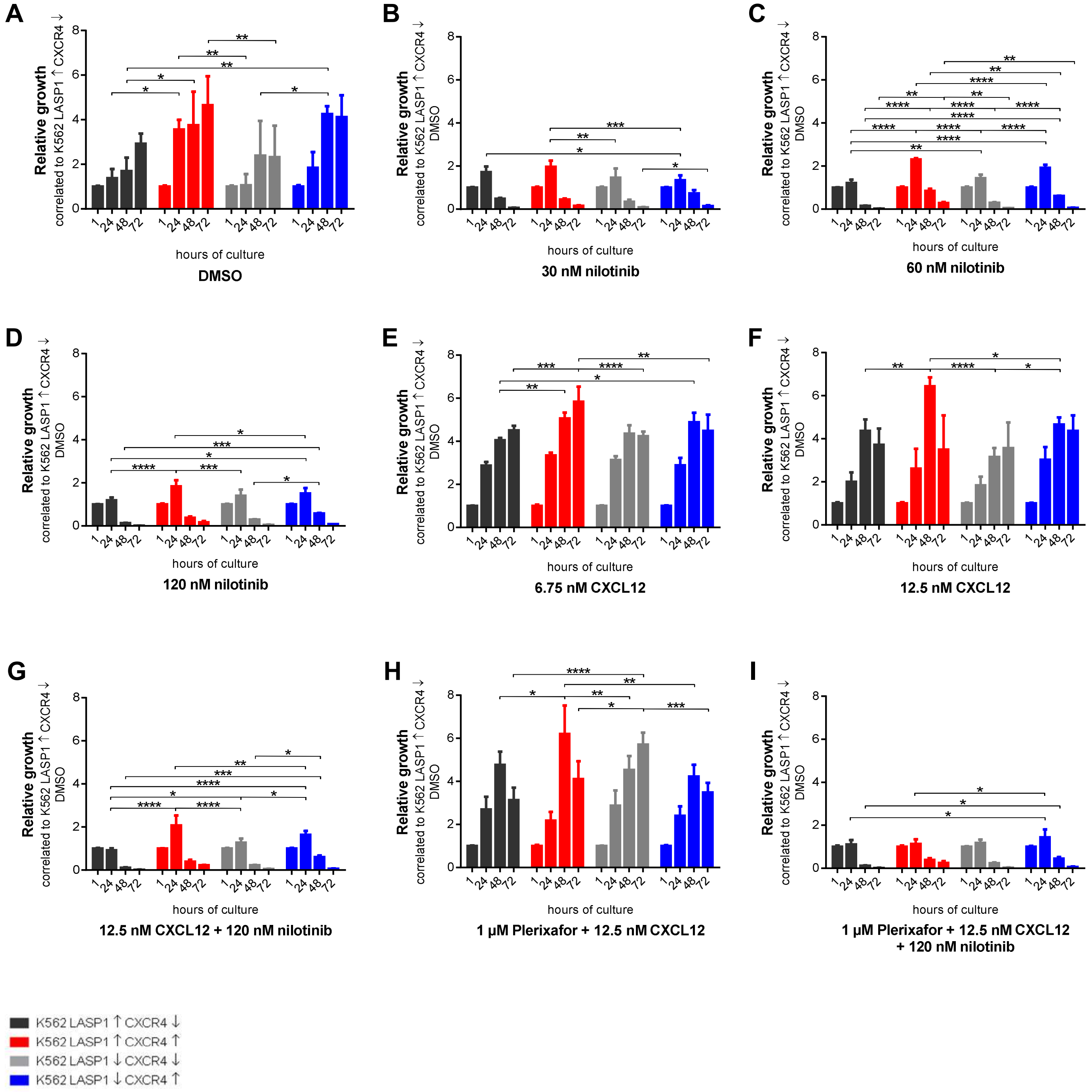

Supplemental Figure S2: Apoptosis

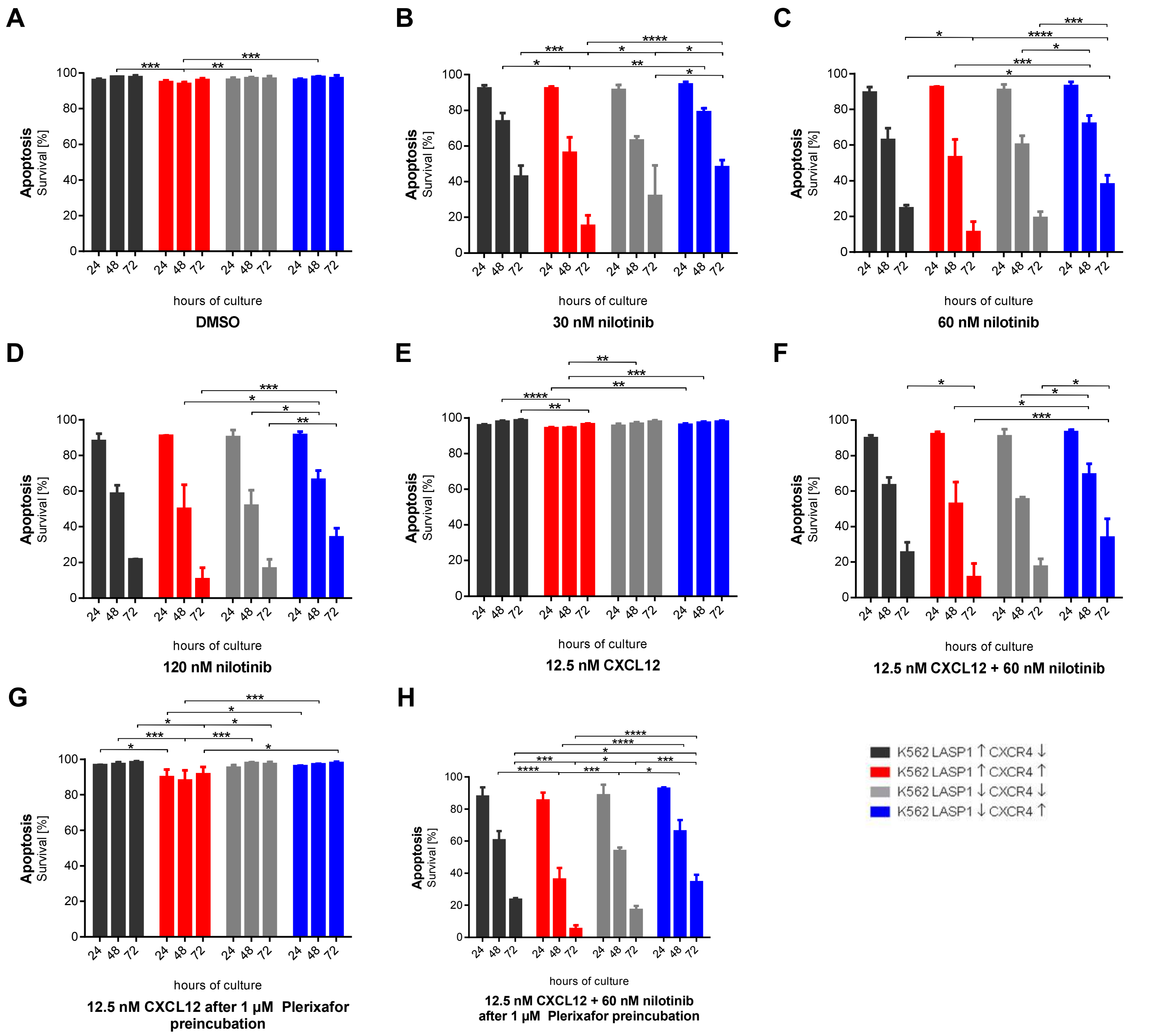

Supplemental Figure S3: Cell Cycle Analysis

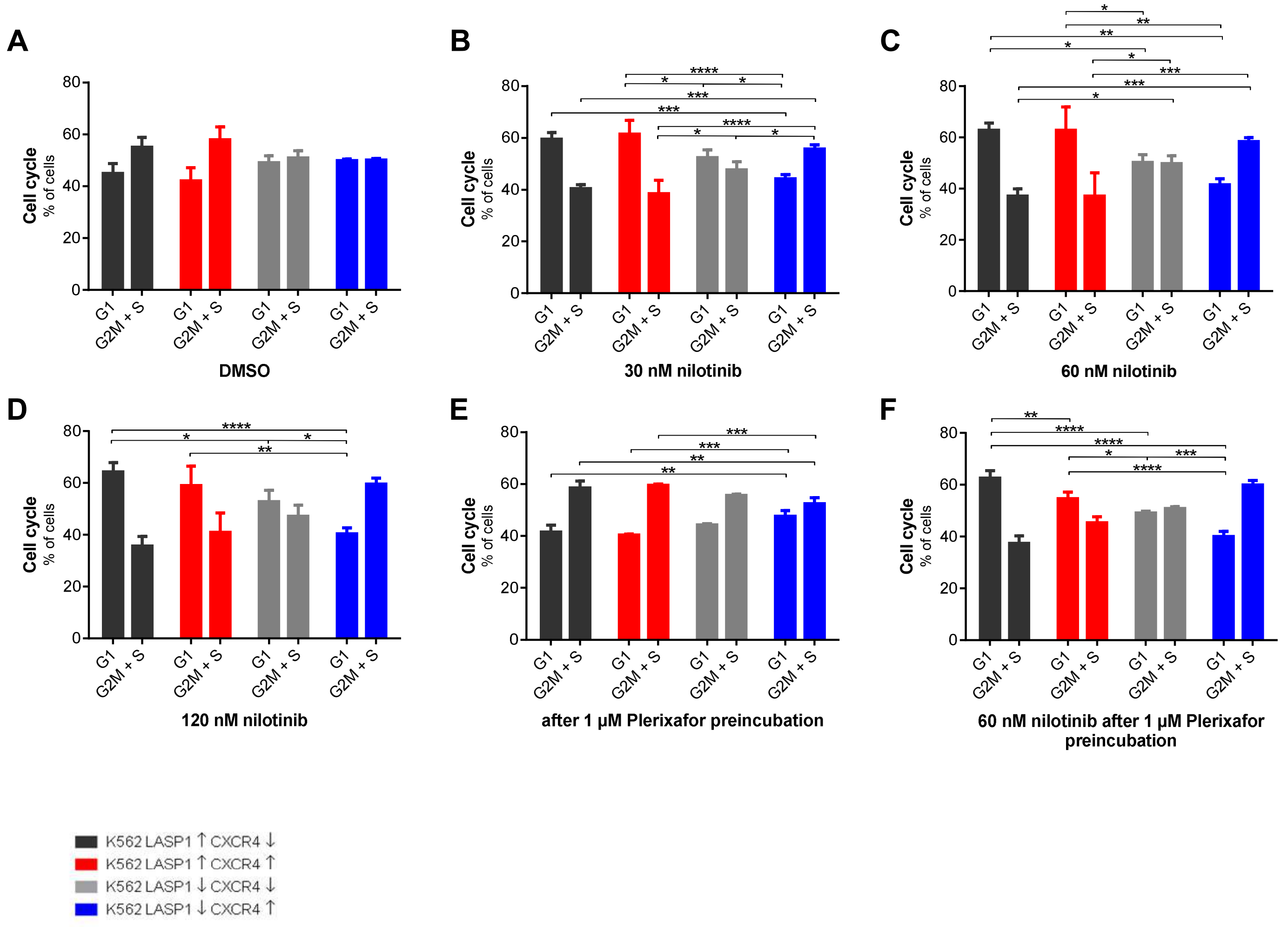

Supplemental Figure S4: Migration

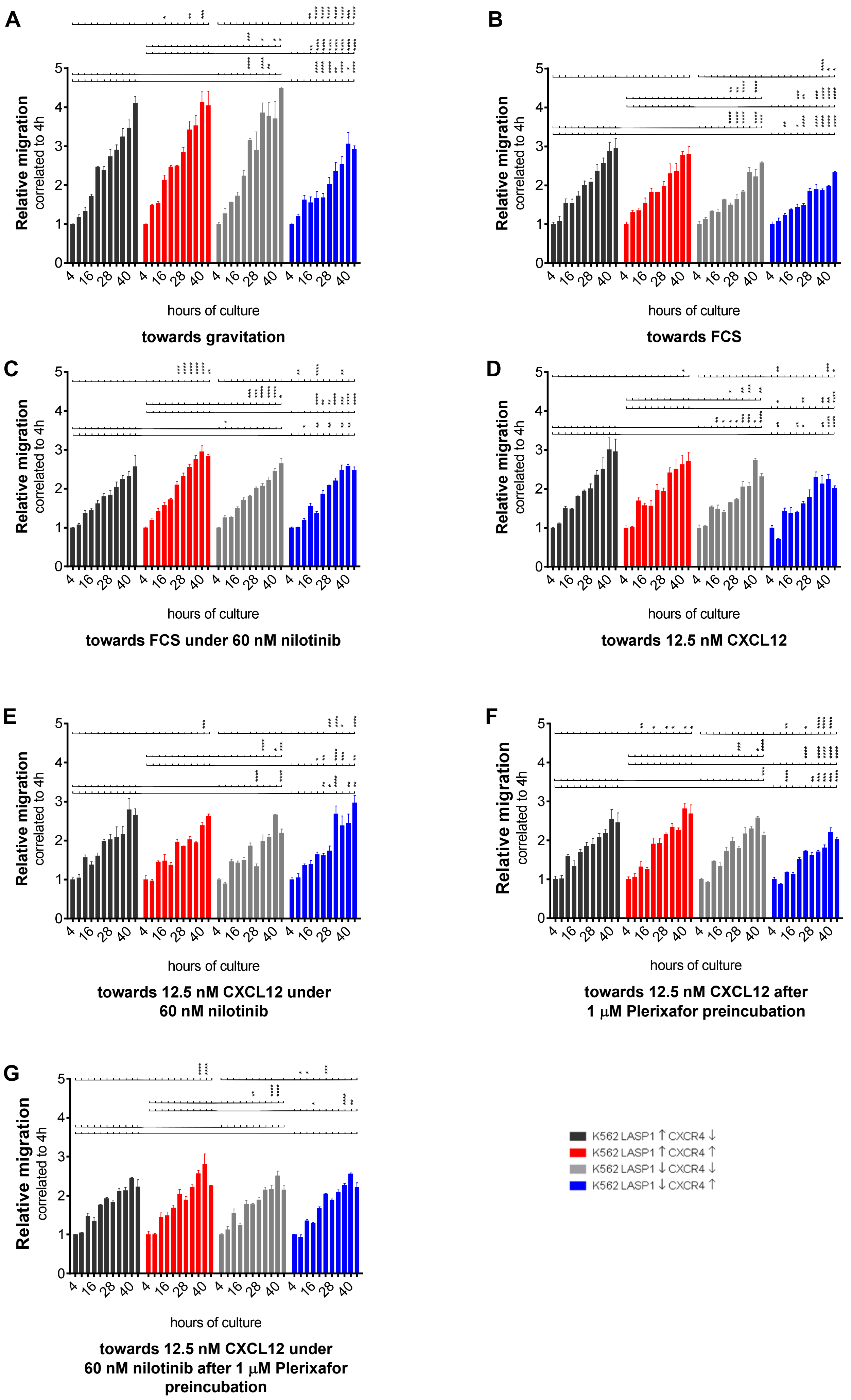

Supplemental Figure S5: Cytokine Levels

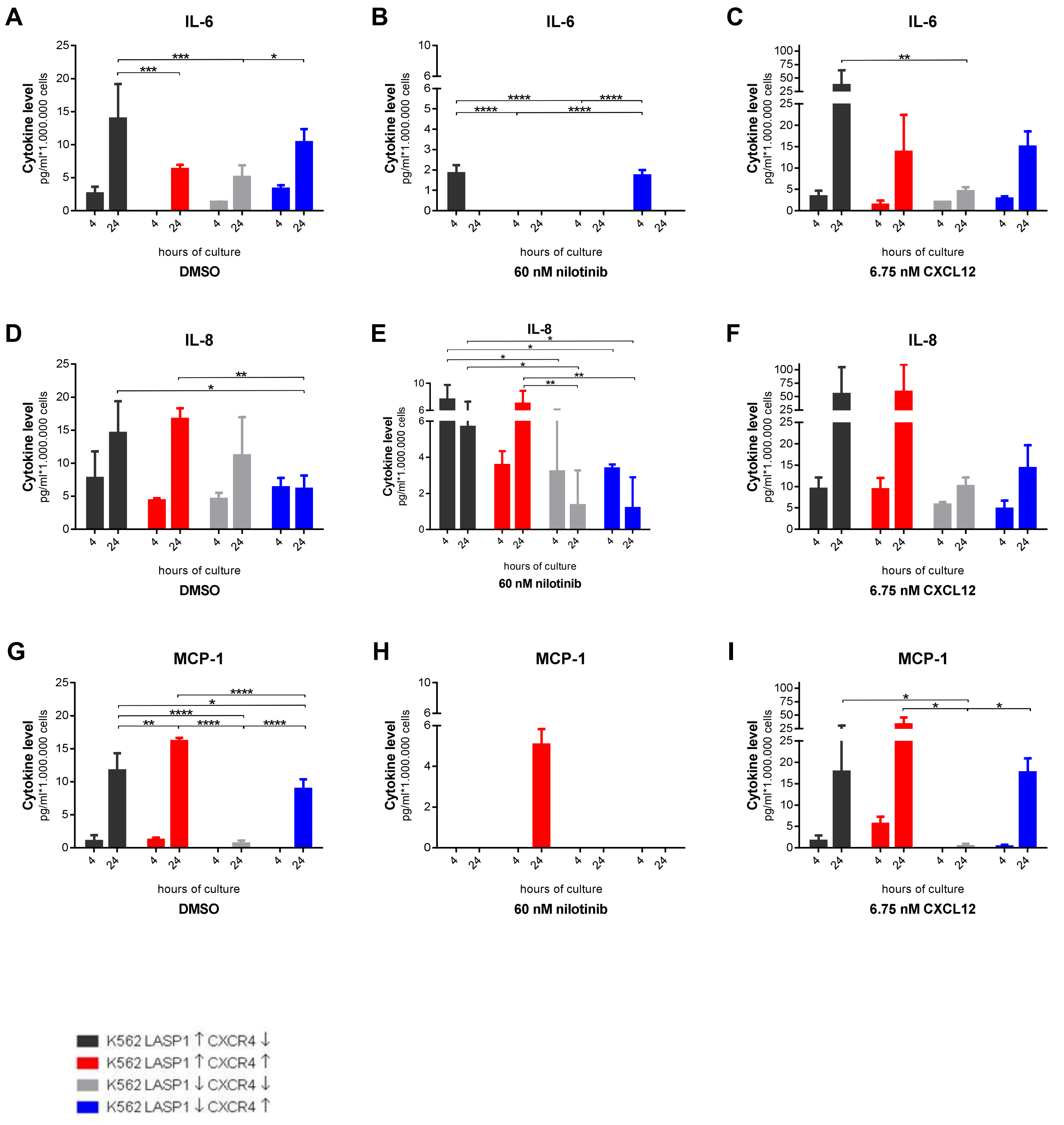

Supplemental Figure S6: Relative Expression

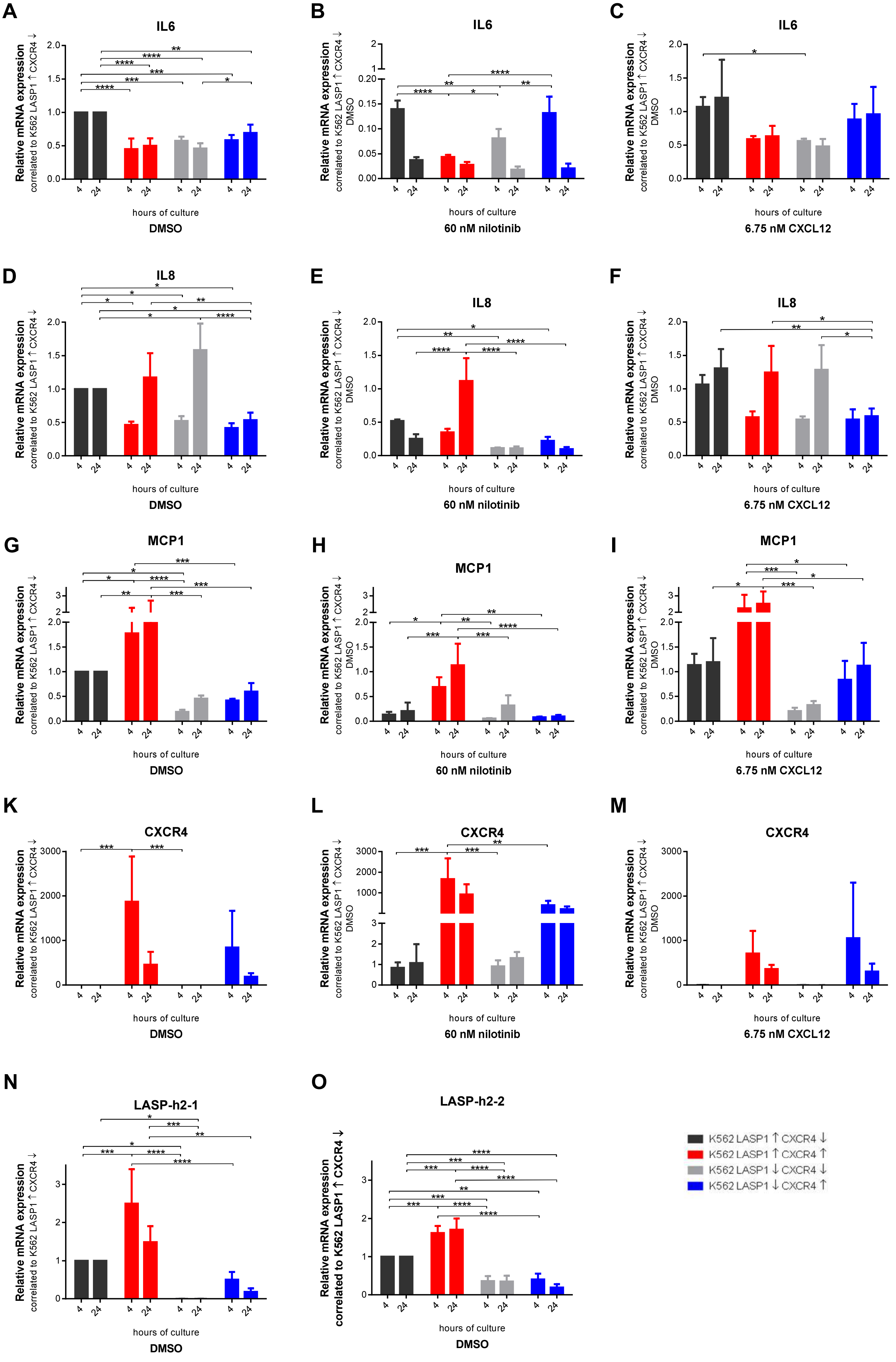

Supplemental Figure S7: Degranulation

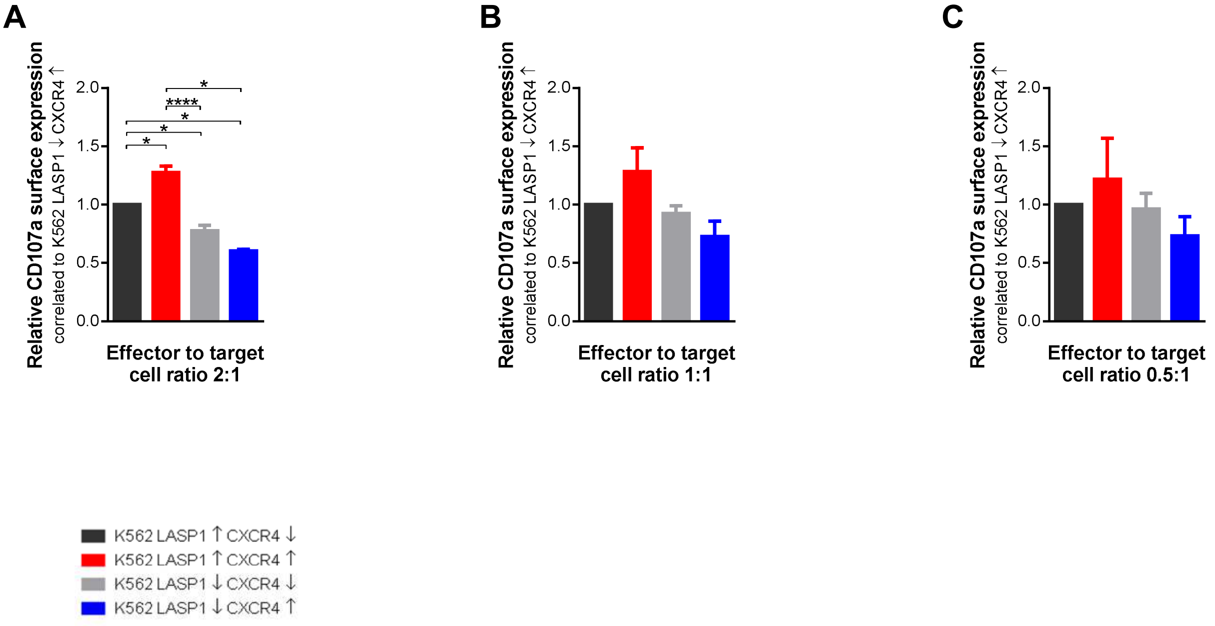

Supplemental Figure S8: Influence on Cytoskeleton Proteins

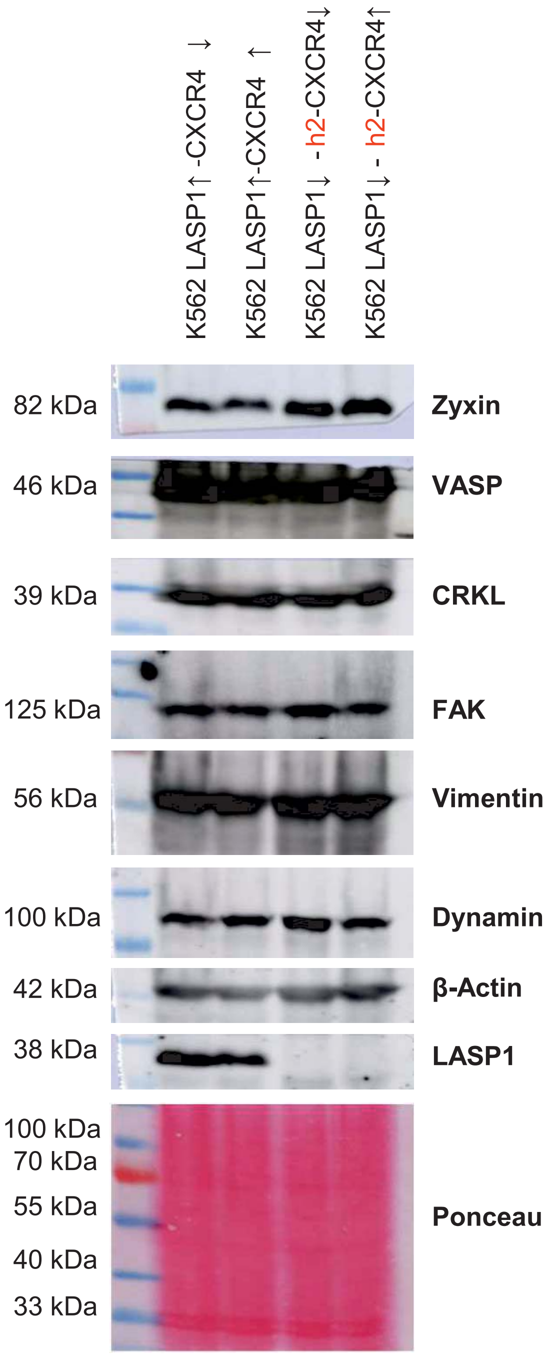

Supplemental Figure S9: Illustration of LASP1 Double Nicking by CRIPR/Cas9

```
5'CACCATGGTGGCGGACACCCCGGAAAACCTTCGCCTCAAGCAACAGAGTGAGCTCCAGAGTCAGGTGCGCTACAAGGAGGAG3'
3'GTGGTACCACCGCCTGTGGGGCCTTTTGGAAGCGGAGTTCGTTGTCTCACTCGAGGTCTCAGTCCACGCGATGTTCCTCCTC5'
```

Supplemental Figure S10: Relative LASP2 Expression

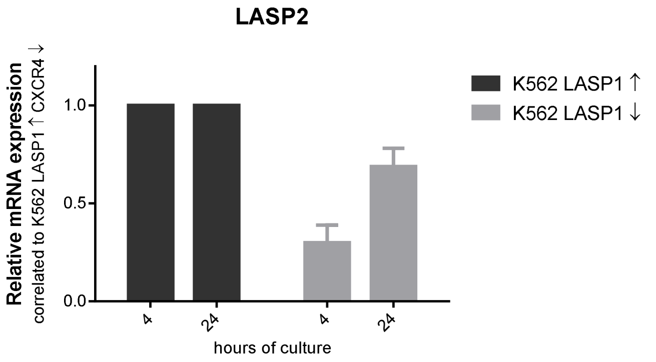

Supplement: Supplementary file 2 [file JCMM-24-2942-s002.pdf]
