# Supplementary material for: Knockout of LASP1 in CXCR4 expressing CML cells promotes cell persistence, proliferation and TKI resistance
Source: J Cell Mol Med. 2020 Jan 19;24(5):2942–55. doi: 10.1111/jcmm.14910 (PMC7077607; doi:10.1111/jcmm.14910)
Supplement: Supplementary file 3 [file JCMM-24-2942-s003.pdf]

Supplemental Table 1: Primer Sequences for qRT-PCR

	Primer sequences (5' - 3')		
Gene	Forward	Reverse	annealing temperature [°C]
CXCR4	AGG GGA TCA GTA TAT ACA CTT CAG A	GAG GAT CTT GAG GCT GGA CC	62
IL-6	ATT CCA AAG ATG TAG CCG CC	AGT GCC TCT TTG CTG CTT TC	59
IL-8	AGC CTT CCT GAT TTC TGC AG	TGT TGG CGC AGT GTG GTC	59
LASP1	GAA GAA GCC CTA CTG CA	TGA TCT GGT CCT GGG TCT TC	62
LASP1-NIC-h2-1	CAC TGA AGT CCT CCT GTG GG	CTT GAG GCG AAG GTT TTC CG	61
LASP1-NIC-h2-2	ACA GAG TGA GCT CCA GAG TC	ATT TCA GCA CCT TGG CCT TG	61
LASP2	GCG GAA AAG TCG TGT ATC CC	CGT GGT GAA GGA CTG CTT C	60
MCP-1	AAA GTC TCT GCC GCC CTT C	ACT TGC TGC TGG TGA TTC TTC	60

Supplemental Table 2: GSEA: Top 50 results from GSEA for LASP1 co- and antiregulated gene sets in imatinib nonresponders and responders

Nonresponders

LASP1-coregulated NAME	NES	NOM p-val	FDR q-val	FWER p-val	LASP1-antiregulated NAME	NES	NOM p-val	FDR q-val	FWER p-val
KEGG OLFACTORY TRANSDUCTION	2.4198	0.0000	0.0011	0.0010	REACTOME RESPIRATORY ELECTRON TRANSPORT	-2.5147	0.0000	0.0000	0.0000
REACTOME OLFACTORY SIGNALING PATHWAY	2.4164	0.0000	0.0006	0.0010	REACTOME RESPIRATORY ELECTRON TRANSPORT ATP SYNTHESIS BY CHEMIOSMOTIC COUPLING AND HEAT PRODUCTION BY UNCOUPLING PROTEINS	-2.4482	0.0000	0.0000	0.0000
BIOCARTA IL2 PATHWAY	2.1150	0.0000	0.0298	0.0730	ROSS AML OF FAB M7 TYPE	-2.4475	0.0000	0.0000	0.0000
PID REELIN PATHWAY	2.0991	0.0000	0.0297	0.0970	MOOTHA VOXPHOS	-2.3493	0.0000	0.0000	0.0000
DUNNE TARGETS OF AML1 MTG8 FUSION UP	2.0786	0.0000	0.0318	0.1270	KEGG PARKINSONS DISEASE	-2.2926	0.0000	0.0003	0.0020
REACTOME INCRETIN SYNTHESIS SECRETION AND INACTIVATION	2.0478	0.0000	0.0416	0.1930	BILANGES SERUM AND RAPAMYCIN SENSITIVE GENES	-2.2893	0.0000	0.0003	0.0020
PID EPHA FWDPATHWAY	2.0339	0.0000	0.0434	0.2330	WELCH GATA1 TARGETS	-2.2701	0.0000	0.0006	0.0050
BIOCARTA IL2RB PATHWAY	2.0125	0.0000	0.0496	0.2910	REACTOME PEPTIDE CHAIN ELONGATION	-2.2679	0.0000	0.0006	0.0060
HAHTOLA CTCL PATHOGENESIS	1.9883	0.0000	0.0616	0.3860	CHNG MULTIPLE MYELOMA HYPERPLOID UP	-2.2503	0.0000	0.0011	0.0110
RICKMAN HEAD AND NECK CANCER C	1.9776	0.0000	0.0630	0.4200	REACTOME RNA POL I TRANSCRIPTION	-2.2364	0.0000	0.0014	0.0160
BIOCARTA BCR PATHWAY	1.9740	0.0000	0.0595	0.4340	PECE MAMMARY STEM CELL UP	-2.2150	0.0000	0.0017	0.0210
OUYANG PROSTATE CANCER PROGRESSION UP	1.9693	0.0000	0.0576	0.4510	REACTOME INFLUENZA VIRAL RNA TRANSCRIPTION AND REPLICATION	-2.2114	0.0000	0.0016	0.0220
PLASARI NFIC TARGETS BASAL UP	1.9666	0.0019	0.0547	0.4610	LI DCP2 BOUND MRNA	-2.1969	0.0000	0.0022	0.0320
PID HEDGEHOG GLI PATHWAY	1.9639	0.0020	0.0529	0.4740	REACTOME TRANSCRIPTION	-2.1895	0.0000	0.0023	0.0370
PID TXA2PATHWAY	1.9595	0.0000	0.0516	0.4890	REACTOME RNA POL I RNA POL III AND MITOCHONDRIAL TRANSCRIPTION	-2.1813	0.0000	0.0023	0.0400
REACTOME SYNTHESIS SECRETION AND INACTIVATION OF GLP1	1.9528	0.0020	0.0517	0.5170	REACTOME FORMATION OF TRANSCRIPTION COUPLED NER TC NER REPAIR COMPLEX	-2.1798	0.0000	0.0023	0.0430
PID AR PATHWAY	1.9354	0.0019	0.0598	0.5930	CHEN HOXA5 TARGETS 9HR UP	-2.1770	0.0000	0.0023	0.0450
MCCLUNG DELTA FOSB TARGETS 8WK	1.9340	0.0000	0.0572	0.5980	REACTOME FORMATION OF THE HIV1 EARLY ELONGATION COMPLEX	-2.1702	0.0000	0.0024	0.0500
MCBRYAN PUBERTAL BREAST 6 7WK UP	1.9297	0.0000	0.0575	0.6190	KEGG RIBOSOME	-2.1619	0.0000	0.0025	0.0540
BIOCARTA PDGF PATHWAY	1.9266	0.0000	0.0565	0.6320	WONG MITOCHONDRIA GENE MODULE	-2.1577	0.0000	0.0027	0.0590
BIOCARTA CDC42RAC PATHWAY	1.9228	0.0000	0.0564	0.6480	MANN RESPONSE TO AMIFOSTINE UP	-2.1573	0.0000	0.0025	0.0590
TAKEDA TARGETS OF NUP98 HOXA9 FUSION 8D DN	1.9098	0.0000	0.0637	0.7090	REACTOME RNA POL I PROMOTER OPENING	-2.1335	0.0000	0.0035	0.0850
BIOCARTA IL6 PATHWAY	1.9090	0.0018	0.0615	0.7150	REACTOME 3 UTR MEDIATED TRANSLATIONAL REGULATION	-2.1322	0.0000	0.0034	0.0860
SCHAEFFER SOX9 TARGETS IN PROSTATE DEVELOPMENT DN	1.9088	0.0000	0.0591	0.7150	REACTOME RNA POL III CHAIN ELONGATION	-2.1151	0.0000	0.0039	0.1010
LEE DIFFERENTIATING T LYMPHOCYTE	1.9081	0.0000	0.0571	0.7150	STARK PREFRONTAL CORTEX 22Q11 DELETION DN	-2.1004	0.0000	0.0048	0.1290
WANG IMMORTALIZED BY HOXA9 AND MEIS1 UP	1.8994	0.0000	0.0603	0.7590	GRAHAM CML QUIESCENT VS NORMAL QUIESCENT UP	-2.0907	0.0000	0.0052	0.1450
REACTOME REGULATION OF SIGNALING BY CBL	1.8906	0.0020	0.0642	0.7930	KEGG HUNTINGTONS DISEASE	-2.0851	0.0000	0.0053	0.1540
MCBRYAN PUBERTAL BREAST 5 6WK DN	1.8831	0.0000	0.0669	0.8160	REACTOME FORMATION OF RNA POL II ELONGATION COMPLEX	-2.0793	0.0000	0.0054	0.1600
KAMIKUBO MYELOID CEBPA NETWORK	1.8815	0.0000	0.0661	0.8270	REACTOME ABORTIVE ELONGATION OF HIV1 TRANSCRIPT IN THE ABSENCE OF TAT	-2.0743	0.0022	0.0056	0.1740
BIOCARTA EGF PATHWAY	1.8802	0.0019	0.0647	0.8320	REACTOME RNA POL II TRANSCRIPTION PRE INITIATION AND PROMOTER OPENING	-2.0701	0.0000	0.0057	0.1800
KEGG VIBRIO CHOLERAE INFECTION	1.8779	0.0019	0.0639	0.8410	REACTOME PACKAGING OF TELOMERE ENDS	-2.0659	0.0000	0.0057	0.1900
LIU NASOPHARYNGEAL CARCINOMA	1.8745	0.0000	0.0641	0.8480	KEGG OXIDATIVE PHOSPHORYLATION	-2.0652	0.0000	0.0056	0.1920
PID IL2 STAT5 PATHWAY	1.8737	0.0019	0.0626	0.8500	REACTOME RNA POL II PRE TRANSCRIPTION EVENTS	-2.0590	0.0000	0.0061	0.2110
PID MAPK TRK PATHWAY	1.8666	0.0019	0.0656	0.8720	TAKAO RESPONSE TO UVB RADIATION UP	-2.0416	0.0000	0.0071	0.2530
BIOCARTA PYK2 PATHWAY	1.8653	0.0020	0.0648	0.8750	KEGG RNA POLYMERASE	-2.0145	0.0000	0.0094	0.3270
BILBAN B CLL LPL UP	1.8650	0.0000	0.0632	0.8750	REACTOME TCA CYCLE AND RESPIRATORY ELECTRON TRANSPORT	-2.0138	0.0000	0.0093	0.3290
BIOCARTA BIOPEPTIDES PATHWAY	1.8578	0.0000	0.0665	0.8920	MOREAUX MULTIPLE MYELOMA BY TACI DN	-1.9942	0.0000	0.0111	0.3900
REACTOME AMINO ACID AND OLIGOPEPTIDE SLC TRANSPORTERS	1.8532	0.0036	0.0683	0.9060	REACTOME ACTIVATION OF THE MRNA UPON BINDING OF THE CAP BINDING COMPLEX AND EIFS AND SUBSEQUENT BINDING TO 43S	-1.9889	0.0000	0.0115	0.4130
VERNOCHET ADIPOGENESIS	1.8517	0.0020	0.0677	0.9120	REACTOME RNA POL III TRANSCRIPTION TERMINATION	-1.9794	0.0000	0.0127	0.4500
BIOCARTA MET PATHWAY	1.8482	0.0000	0.0687	0.9180	DAZARD UV RESPONSE CLUSTER G2	-1.9705	0.0042	0.0138	0.4790
HINATA NFKB TARGETS KERATINOCYTE DN	1.8459	0.0000	0.0688	0.9230	SARTIPY BLUNTED BY INSULIN RESISTANCE DN	-1.9501	0.0021	0.0169	0.5580
REACTOME ANTIGEN ACTIVATES B CELL RECEPTOR LEADING TO GENERATION OF SECOND MESSENGERS	1.8346	0.0000	0.0758	0.9420	GAZDA DIAMOND BLACKFAN ANEMIA MYELOID DN	-1.9304	0.0020	0.0203	0.6440
PID A6B1 A6B4 INTEGRIN PATHWAY	1.8331	0.0000	0.0750	0.9440	REACTOME ELONGATION ARREST AND RECOVERY	-1.9268	0.0000	0.0204	0.6550
KEGG STARCH AND SUCROSE METABOLISM	1.8316	0.0000	0.0746	0.9460	REACTOME MRNA CAPPING	-1.9135	0.0000	0.0230	0.7010
FINAK BREAST CANCER SDPP SIGNATURE	1.8316	0.0000	0.0729	0.9460	REACTOME RNA POL III TRANSCRIPTION INITIATION FROM TYPE 2 PROMOTER	-1.9114	0.0020	0.0229	0.7060
PID MET PATHWAY	1.8249	0.0000	0.0761	0.9560	YAO TEMPORAL RESPONSE TO PROGESTERONE CLUSTER 13	-1.9084	0.0000	0.0229	0.7140
GENTLES LEUKEMIC STEM CELL DN	1.8177	0.0019	0.0802	0.9660	DACOSTA UV RESPONSE VIA ERCC3 COMMON UP	-1.9081	0.0000	0.0225	0.7140
KEGG FC GAMMA R MEDIATED PHAGOCYTOSIS	1.8174	0.0000	0.0788	0.9660	REACTOME INFLUENZA LIFE CYCLE	-1.9024	0.0000	0.0235	0.7430
PID P38 MK2 PATHWAY	1.8172	0.0037	0.0774	0.9660	NIKOLSKY BREAST CANCER 16P13 AMPLICON	-1.8984	0.0000	0.0241	0.7590
REACTOME RAS ACTIVATION UOPN CA2 INFLUX THROUGH NMDA RECEPTOR	1.8162	0.0057	0.0765	0.9660	KIM WT1 TARGETS DN	-1.8941	0.0000	0.0246	0.7670

Supplemental Table 2: GSEA: Top 50 results from GSEA for LASP1 co- and antiregulated gene sets in imatinib nonresponders and responders

Responders

LASP1-coregulated NAME	NES	NOM p-val	FDR q-val	FWER p-val	LASP1-antiregulated NAME	NES	NOM p-val	FDR q-val	FWER p-val
KAMIKUBO MYELOID CEBPA NETWORK	2.5782	0.0000	0.0000	0.0000	GRAHAM CML DIVIDING VS NORMAL QUIESCENT UP	-3.1167	0.0000	0.0000	0.0000
TAKEDA TARGETS OF NUP98 HOXA9 FUSION 16D DN	2.5665	0.0000	0.0000	0.0000	MOREAUX MULTIPLE MYELOMA BY TACI DN	-3.0600	0.0000	0.0000	0.0000
TAKEDA TARGETS OF NUP98 HOXA9 FUSION 8D DN	2.5657	0.0000	0.0000	0.0000	GARY CD5 TARGETS DN	-3.0426	0.0000	0.0000	0.0000
GRAHAM CML QUIESCENT VS NORMAL QUIESCENT DN	2.5532	0.0000	0.0000	0.0000	MANALO HYPOXIA DN	-3.0398	0.0000	0.0000	0.0000
PID IL8 CXCR1 PATHWAY	2.5319	0.0000	0.0000	0.0000	EPPERT PROGENITOR	-2.9954	0.0000	0.0000	0.0000
PARK TRETINOIN RESPONSE AND PML RARA FUSION	2.4329	0.0000	0.0000	0.0000	CHIANG LIVER CANCER SUBCLASS UNANNOTATED DN	-2.9542	0.0000	0.0000	0.0000
PID IL8 CXCR2 PATHWAY	2.4311	0.0000	0.0000	0.0000	ROSTY CERVICAL CANCER PROLIFERATION CLUSTER	-2.9324	0.0000	0.0000	0.0000
JAATINEN HEMATOPOIETIC STEM CELL DN	2.4234	0.0000	0.0000	0.0000	GRAHAM CML QUIESCENT VS NORMAL QUIESCENT UP	-2.9223	0.0000	0.0000	0.0000
BROWN MYELOID CELL DEVELOPMENT UP	2.3451	0.0000	0.0004	0.0030	WONG EMBRYONIC STEM CELL CORE	-2.9113	0.0000	0.0000	0.0000
LEE DIFFERENTIATING T LYMPHOCYTE	2.3345	0.0000	0.0003	0.0030	BENPORATH PROLIFERATION	-2.9110	0.0000	0.0000	0.0000
WINNEPENNINCKX MELANOMA METASTASIS DN	2.3009	0.0000	0.0004	0.0040	WONG MITOCHONDRIA GENE MODULE	-2.9076	0.0000	0.0000	0.0000
HOEBEKE LYMPHOID STEM CELL UP	2.2903	0.0000	0.0007	0.0070	ROSS AML OF FAB M7 TYPE	-2.8934	0.0000	0.0000	0.0000
BOYLAN MULTIPLE MYELOMA C D DN	2.2873	0.0000	0.0006	0.0070	SOTIRIOU BREAST CANCER GRADE 1 VS 3 UP	-2.8875	0.0000	0.0000	0.0000
MARTENS BOUND BY PML RARA FUSION	2.2810	0.0000	0.0006	0.0080	MILI PSEUDOPODIA HAPTOTAXIS UP	-2.8693	0.0000	0.0000	0.0000
OUELLET CULTURED OVARIAN CANCER INVASIVE VS LMP DN	2.2650	0.0000	0.0007	0.0100	SHEN SMARCA2 TARGETS UP	-2.8639	0.0000	0.0000	0.0000
DUNNE TARGETS OF AML1 MTG8 FUSION UP	2.2513	0.0000	0.0009	0.0130	MOOTHA HUMAN MITODB 6 2002	-2.8567	0.0000	0.0000	0.0000
KEGG PHOSPHATIDYLINOSITOL SIGNALING SYSTEM	2.2498	0.0000	0.0009	0.0130	WINNEPENNINCKX MELANOMA METASTASIS UP	-2.8501	0.0000	0.0000	0.0000
SENGUPTA EBNA1 ANTICORRELATED	2.2453	0.0000	0.0008	0.0130	REACTOME TRANSLATION	-2.8262	0.0000	0.0000	0.0000
CHYLA CBFA2T3 TARGETS UP	2.2240	0.0000	0.0015	0.0250	GAZDA DIAMOND BLACKFAN ANEMIA PROGENITOR DN	-2.8239	0.0000	0.0000	0.0000
LEE EARLY T LYMPHOCYTE DN	2.2235	0.0000	0.0014	0.0250	STARK PREFRONTAL CORTEX 22Q11 DELETION DN	-2.8136	0.0000	0.0000	0.0000
KEGG LEUKOCYTE TRANSENDOTHELIAL MIGRATION	2.2126	0.0000	0.0016	0.0300	KEGG SPLICEOSOME	-2.7932	0.0000	0.0000	0.0000
GRAHAM CML DIVIDING VS NORMAL QUIESCENT DN	2.2093	0.0000	0.0016	0.0310	REACTOME INFLUENZA LIFE CYCLE	-2.7800	0.0000	0.0000	0.0000
TONKS TARGETS OF RUNX1 RUNX1T1 FUSION ERYTHROCYTE UP	2.1715	0.0000	0.0032	0.0620	GRAHAM NORMAL QUIESCENT VS NORMAL DIVIDING DN	-2.7786	0.0000	0.0000	0.0000
REACTOME SIGNALING BY RHO GTPASES	2.1692	0.0000	0.0031	0.0630	SCHLOSSER MYC TARGETS REPRESSED BY SERUM	-2.7778	0.0000	0.0000	0.0000
KEGG NATURAL KILLER CELL MEDIATED CYTOTOXICITY	2.1558	0.0000	0.0034	0.0710	REACTOME 3 UTR MEDIATED TRANSLATIONAL REGULATION	-2.7727	0.0000	0.0000	0.0000
ZHENG FOXP3 TARGETS IN T LYMPHOCYTE DN	2.1501	0.0000	0.0036	0.0770	REACTOME INFLUENZA VIRAL RNA TRANSCRIPTION AND REPLICATION	-2.7726	0.0000	0.0000	0.0000
SMID BREAST CANCER NORMAL LIKE UP	2.1497	0.0000	0.0035	0.0780	REACTOME PEPTIDE CHAIN ELONGATION	-2.7634	0.0000	0.0000	0.0000
REACTOME GASTRIN CREB SIGNALLING PATHWAY VIA PKC AND MAPK	2.1400	0.0000	0.0038	0.0890	SHEDDEN LUNG CANCER POOR SURVIVAL A6	-2.7585	0.0000	0.0000	0.0000
VERNOCHET ADIPOGENESIS	2.1361	0.0000	0.0041	0.0990	MOOTHA MITOCHONDRIA	-2.7369	0.0000	0.0000	0.0000
REACTOME G ALPHA Q SIGNALLING EVENTS	2.1358	0.0000	0.0039	0.0990	KEGG RIBOSOME	-2.7181	0.0000	0.0000	0.0000
PID TCR PATHWAY	2.1253	0.0000	0.0041	0.1070	ZHANG TLX TARGETS 60HR DN	-2.7161	0.0000	0.0000	0.0000
MIKKELSEN ES ICP WITH H3K4ME3 AND H3K27ME3	2.1157	0.0000	0.0046	0.1230	ZHANG TLX TARGETS DN	-2.6770	0.0000	0.0000	0.0000
LIM MAMMARY LUMINAL PROGENITOR UP	2.1008	0.0000	0.0051	0.1400	REACTOME RESPIRATORY ELECTRON TRANSPORT ATP SYNTHESIS BY CHEMIOSMOTIC COUPLING AND HEAT PRODUCTION BY UNCOUPLING PROTEINS	-2.6661	0.0000	0.0000	0.0000
ICHIBA GRAFT VERSUS HOST DISEASE 35D UP	2.0995	0.0000	0.0050	0.1400	REACTOME TCA CYCLE AND RESPIRATORY ELECTRON TRANSPORT	-2.6641	0.0000	0.0000	0.0000
WANG IMMORTALIZED BY HOXA9 AND MEIS1 UP	2.0882	0.0000	0.0059	0.1680	KIM WT1 TARGETS DN	-2.6630	0.0000	0.0000	0.0000
SMID BREAST CANCER RELAPSE IN PLEURA DN	2.0773	0.0000	0.0067	0.1950	REACTOME ACTIVATION OF THE MRNA UPON BINDING OF THE CAP BINDING COMPLEX AND EIFS AND SUBSEQUENT BINDING TO 43S	-2.6628	0.0000	0.0000	0.0000
PYEON CANCER HEAD AND NECK VS CERVICAL DN	2.0767	0.0000	0.0065	0.1950	FARMER BREAST CANCER CLUSTER 2	-2.6593	0.0000	0.0000	0.0000
MCLACHLAN DENTAL CARIES UP	2.0737	0.0000	0.0065	0.2010	REACTOME RNA POL II TRANSCRIPTION PRE INITIATION AND PROMOTER OPENING	-2.6576	0.0000	0.0000	0.0000
REACTOME G ALPHA1213 SIGNALLING EVENTS	2.0731	0.0000	0.0064	0.2010	BERENJENO TRANSFORMED BY RHOA UP	-2.6518	0.0000	0.0000	0.0000
DAVIES MULTIPLE MYELOMA VS MGUS DN	2.0721	0.0000	0.0064	0.2040	CHNG MULTIPLE MYELOMA HYPERPLOID UP	-2.6443	0.0000	0.0000	0.0000
KEGG CHEMOKINE SIGNALING PATHWAY	2.0721	0.0000	0.0062	0.2040	ZHANG TLX TARGETS 36HR DN	-2.6411	0.0000	0.0000	0.0000
GENTLES LEUKEMIC STEM CELL UP	2.0633	0.0000	0.0066	0.2190	REACTOME LATE PHASE OF HIV LIFE CYCLE	-2.6134	0.0000	0.0000	0.0000
PID ALPHA SYNUCLEIN PATHWAY	2.0628	0.0000	0.0065	0.2190	REACTOME RESPIRATORY ELECTRON TRANSPORT	-2.6124	0.0000	0.0000	0.0000
REACTOME IL 3 5 AND GM CSF SIGNALING	2.0607	0.0000	0.0064	0.2220	MALONEY RESPONSE TO 17AAG DN	-2.6121	0.0000	0.0000	0.0000
VALK AML WITH FLT3 ITD	2.0562	0.0018	0.0066	0.2280	RHEIN ALL GLUCOCORTICOID THERAPY DN	-2.6040	0.0000	0.0000	0.0000
MCBRYAN PUBERTAL BREAST 6 7WK UP	2.0536	0.0000	0.0066	0.2320	REACTOME SRP DEPENDENT COTRANSLATIONAL PROTEIN TARGETING TO MEMBRANE	-2.5998	0.0000	0.0000	0.0000
VERHAAK GLIOBLASTOMA MESENCHYMAL	2.0506	0.0000	0.0069	0.2470	KOBAYASHI EGFR SIGNALING 24HR DN	-2.5970	0.0000	0.0000	0.0000
VERHAAK AML WITH NPM1 MUTATED UP	2.0488	0.0000	0.0069	0.2540	FISCHER G2 M CELL CYCLE	-2.5961	0.0000	0.0000	0.0000
THEILGAARD NEUTROPHIL AT SKIN WOUND DN	2.0478	0.0000	0.0069	0.2580	DING LUNG CANCER EXPRESSION BY COPY NUMBER	-2.5880	0.0000	0.0000	0.0000
TAKEDA TARGETS OF NUP98 HOXA9 FUSION 10D DN	2.0426	0.0000	0.0071	0.2720	REACTOME RNA POL II PRE TRANSCRIPTION EVENTS	-2.5792	0.0000	0.0000	0.0000
